# Halogen‐Bond Coupled Halogenated‐π‐Conjugation Enables Giant Birefringence in Hydrogen‐Bonded Organic Frameworks

**DOI:** 10.1002/advs.202600006

**Published:** 2026-01-14

**Authors:** Miao‐Bin Xu, Yun‐Xia Hu, Ming‐Chang Wang, Jia‐Jia Li, Jia‐Min Lian, Jin Chen, Ke‐Zhao Du

**Affiliations:** ^1^ Fujian Provincial Key Laboratory of Advanced Materials Oriented Chemical Engineering College of Chemistry and Material Science Fujian Normal University Fuzhou P. R. China; ^2^ State Key Laboratory of Structural Chemistry Fujian Institute of Research on the Structure of Matter Chinese Academy of Sciences Fuzhou P. R. China

**Keywords:** birefringence, halogen bond, halogenated‐π‐conjugation, structure‐property relationships, supramolecular framework

## Abstract

Birefringent crystals are essential optical materials for modulating and detecting the polarization state of light. However, achieving large optical anisotropy remains a significant challenge, mainly because it is difficult to rationally align anisotropic fundamental building blocks (FBBs) to maximize their collective contribution. Here, we propose a halogen‐bond (XB) coupled halogenated‐π‐conjugation strategy. Using hydrogen‐bonded pyridinedicarboxylic acid as a template, we synthesized **XOF‐1**, **Hybrid‐XOF‐1**, and **XB‐HOF‐1** through decarboxylation‐halogenation reactions. All three compounds are built from 0D halogen‐bonded FBBs and extended by halogen and/or hydrogen bonds. Notably, all three compounds exhibit large birefringence (0.69–0.97), with **XB‐HOF‐1** reaching Δn = 0.97 @ 546 nm, over 80 times that of the commercial benchmark MgF_2_ and the highest value reported for any pyridine‐containing system. Theoretical calculations and structural analyses reveal that this exceptional performance originates from the synergistic combination of maximized in‐plane anisotropy, induced by collinear arrangement of N···I‐Cl halogen bonds along one crystallographic axis, and minimized out‐of‐plane polarizability, induced by coplanar alignment of halogenated π‐conjugated planes perpendicular to another axis. Our work establishes an effective strategy for pushing the boundaries of optical anisotropy and offers a new blueprint for the rational design of high‐performance birefringent materials.

## Graphical abstract

The perfect crystal packing achieved via the halogen‐bond (XB) coupled halogenated‐π‐conjugation strategy effectively induces an ultrahigh birefringence (Δn = 0.97), which exhibits promising potential in polarization control and phase modulation.

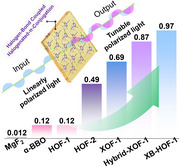

## 1 Introduction

Birefringent crystals are essential components in modern optical technologies, performing critical functions such as polarization control, phase modulation, and beam steering in applications spanning optical communications, laser engineering, and high‐precision instrumentation [1, 2, 3, 4, 5]. However, the performance of current commercial materials, including MgF_2_ (Δn ≈ 0.012), α‐BaB_2_O_4_ (α‐BBO, Δn ≈ 0.12 @ 532 nm), CaCO_3_ (Δn ≈ 0.17 @ 532 nm), and YVO_4_ (Δn ≈ 0.23 @ 532 nm), is often constrained by their moderate birefringence [6, 7, 8, 9, 10, 11]. This limitation presents a significant bottleneck for the further miniaturization and performance enhancement of optical devices. Consequently, the discovery of new materials that exhibit giant birefringence (Δn > 0.3) while maintaining transparency in the demanding ultraviolet (UV) spectral region has emerged as an active research direction in materials chemistry [12, 13, 14, 15, 16, 17, 18, 19].

The magnitude of a crystal's macroscopic birefringence is mainly determined by the polarizability anisotropy (Δα) of its functional building blocks (FBBs) and their spatial arrangement within the crystal lattice [20, 21]. Planar π‐conjugated systems, such as the inorganic groups [CO_3_]^2−^ and [BO_3_]^3−^, or organic N‐heterocycles like cyanurates, are highly attractive FBBs because of their large Δα [22, 23, 24, 25, 26, 27]. To get the highest macroscopic birefringence, achieving an optimal, ideally coplanar, alignment of these anisotropic units is essential [28]. So, strategies using extended π‐conjugation within rigid frameworks have led to materials with ultrahigh birefringence, such as polycyanurates like Cd(H_2_C_6_N_7_O_3_)_2_·8H_2_O (Δn = 0.60 @ 550 nm) [29], K_2_HC_9_N_13_·3H_2_O (0.87 @ 550 nm) [30], Rb_3_[C_6_N_7_(NCN)_3_]·3H_2_O (Δn = 0.60 @ 546 nm) [31], and (MLE)_2_(GAM)_2_PbI_9_ (0.65 @ 550 nm) [32]. Besides, introducing a second type of FBB can also produce crystals with birefringence exceeding 0.5, especially for pyridine and its derivatives (Table S1) [33, 34, 35, 36, 37, 38, 39, 40, 41, 42, 43, 44]. These molecules can be protonated and then combined with another π‐conjugated anion through hydrogen bonds and electrostatic interactions, such as [HPyClB(OH)_2_]·(NO_3_) (Δn = 0.533 @ 546 nm) [34] and Na_2_(4‐HPyH)_2_(PTS)·H_2_O (Δn = 0.811 @ 546 nm) [35]. Moreover, pyridine derivatives can also lose a proton and coordinate with SCALP‐type cations such as Sb^3+^, which can further increase birefringence. For example, (C_10_H_6_NO_2_)_2_SbF (0.87 @ 546 nm) shows the highest birefringence among compounds containing Py‐rings [36]. However, like 2D van der Waals crystals (usually featuring giant out‐of‐plane anisotropy in infrared spectral), such as *h*‐BN, α‐MoO_3_, ReS_2_, and black phosphorus [9, 12, 45, 46, 47, 48, 49, 50, 51, 52, 53, 54], these birefringent crystals dominated by planar π‐conjugated units also face the problem of insufficient in‐plane optical anisotropy, which limits their further application in planar photonic devices [1].

Linear units aligned parallel along a principal axis, such as IX_2_^−^ (X = Cl, Br, I), HgX_2_^−^ (X = Cl, Br, I), and Hg_2_O_2_^2−^, can ensure that the optical axis lies in the cleavage plane, thus showing significant in‐plane optical anisotropy between the chain direction and the interchain direction [1, 6, 9, 12, 41, 43]. So, recently, combining linear polyhalide anions with planar π‐conjugated groups in solution‐processable crystals has become a promising method. Zhao et al. reported C_3_H_8_N_6_I_6_·3H_2_O [1], where melamine cations are arranged coplanar and I_3_^−^ anions are distributed collinearly. The two types of anisotropic groups cooperatively align in the crystal, so the out‐of‐plane and in‐plane polarizability anisotropy can effectively add up, resulting in a maximum birefringence of 2.8. However, the narrow HOMO–LUMO gap of I_3_^−^ leads to a small bandgap (1.68 eV) for this crystal, which limits its application in the ultraviolet and visible regions. Similarly, for [H‐4AP][IBr_2_] [41] reported by OK's group, although it is the first pyridine crystal with birefringence exceeding 0.8, structure‐property analysis shows that its optical anisotropy mainly comes from the linearly arranged IBr_2_^−^ anions, while the π‐conjugated pyridine ring actually contributes negatively to birefringence. The reason is the large dihedral angle (about 69°) between the IBr_2_^−^ unit and the pyridine plane, which makes it difficult for the two to work together to build coplanar anisotropy. Three‐center four‐electron halogen bonds (XBs), such as N···I‐O, represent another class of linear FBBs [55, 56, 57, 58, 59, 60, 61]. Compared to weak hydrogen bonds, XBs can more effectively enforce a coplanar arrangement with the π‐system. This advantage is shown by I^+^(C_6_H_4_NO_2_)^−^ (INA, Δn = 0.778 @ 550 nm) [43], the first XB‐assembled birefringent organic framework. However, the birefringence of INA is still limited, mainly because the N···I‐O type XB has relatively weak polarizability anisotropy, and the carboxyl group on the pyridine ring blocks the optimal collinear alignment of these XB units. How can these limitations be overcome? A promising solution is to combine polyhalide anions and halogen bonds. Specifically, by removing the carboxyl group via halogenation and constructing N···I‐X type XB, it becomes possible to both enhance the polarizability anisotropy of the XB units and rigidly lock the linear anisotropic axis and the pyridine plane into a coplanar geometry.

**FIGURE 1:**
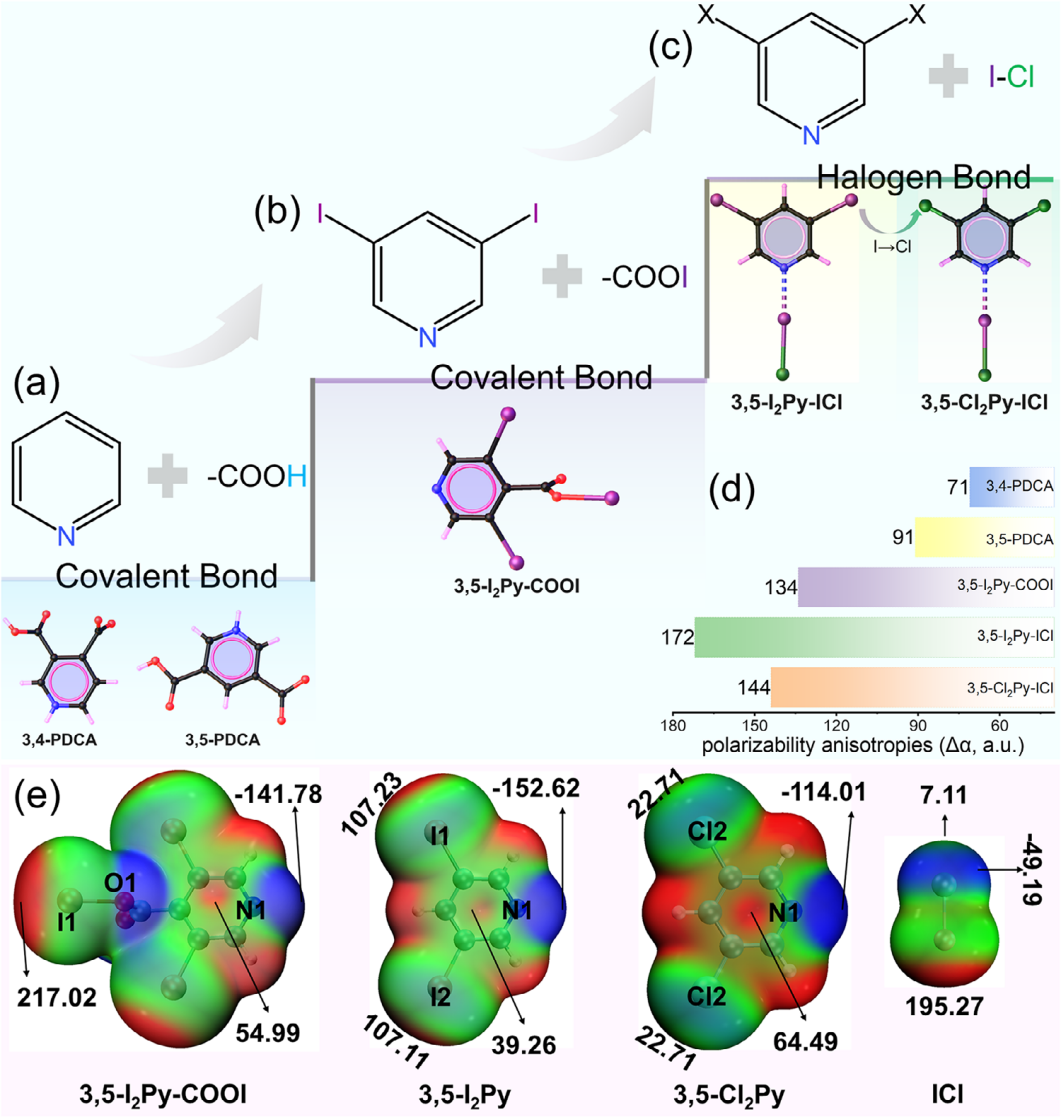
Molecular configuration transformation from 3,4‐PDCA and 3,5‐PDCA (a) to 3,5‐I_2_Py‐ICl (b) and 3,5‐ X_2_Py‐ICl (c); (d) calculated polarizability anisotropy; (e) electrostatic potential (ESP) distribution of individual groups.

Here, we propose a halogen‐bond coupled halogenated‐π‐conjugation strategy based on two synergistic design principles: (1) halogenated‐π‐conjugation, replacing carboxyl substituents on the pyridine ring with halogen atoms to enhance the intrinsic polarizability anisotropy of the π‐conjugated FBBs while eliminating steric hindrance that prevents optimal alignment; and (2) halogen‐bond coupling, using strong, linear N···I‐X halogen bonds to rigidly lock these halogenated π‐conjugated units into a coplanar geometry, thereby maximizing their collective contribution to macroscopic birefringence. And then, we first synthesized crystals of 3,4‐pyridinedicarboxylic acid (3,4‐PDCA) and 3,5‐pyridinedicarboxylic acid (3,5‐PDCA), named **HOF‐1** (hydrogen‐bonded organic framework‐1) and **HOF‐2**, which show birefringence of 0.12 and 0.49 (@ 546 nm), respectively (Figure 1a). Then, a one‐step decarboxylation‐halogenation of **HOF‐1** produced **XOF‐1** (halogen‐bonded organic framework‐1), where 3,5‐I_2_Py‐COOI units form 1D chains through N···I‐O XBs (Figure 1b), achieving a birefringence of 0.69 (@ 546 nm). In parallel, treating **HOF‐2** with ICl yielded **Hybrid‐XOF‐1** (hybrid halogen‐bonded organic framework) and **XB‐HOF‐1** (halogen‐bond‐assembled hydrogen‐bonded organic framework) through two‐step decarboxylation‐halogenation. Their FBBs are adducts (3,5‐I_2_Py‐ICl and 3,5‐Cl_2_Py‐ICl) formed by N···I‐Cl XBs, which further assemble into 1D chains through C‐I···Cl halogen bonds or/and C‐H···Cl hydrogen bonds (Figure 1c). Remarkably, **Hybrid‐XOF‐1** and **XB‐HOF‐1** achieve ultra‐high birefringence of 0.87 and 0.97 (@ 546 nm), respectively. Notably, **XB‐HOF‐1** is the first pyridine system with birefringence exceeding the 0.9 threshold.

## 2 Results and Discussion

### 2.1 Theoretical Investigation of Functional Building Blocks (FBBs)

We first employed density functional theory (DFT) calculations to evaluate the intrinsic optical properties of the designed and synthesized FBBs. The calculated polarizability anisotropies (Δα) for 3,5‐I_2_Py‐COOI, 3,5‐I_2_Py‐ICl, and 3,5‐Cl_2_Py‐ICl are 134, 172, and 144 a.u., respectively (Figure 1d). These values are substantially larger than those of the parent molecules, 3,4‐PDCA (71 a.u.) and 3,5‐PDCA (91 a.u.), confirming that our halogenation strategy effectively enhances the intrinsic optical anisotropy of the FBBs.

To elucidate the directional interactions governing the supramolecular assembly, we analyzed the electrostatic potential (ESP) maps of the FBBs (Figure 1e). The analysis reveals that the negatively charged pyridine nitrogen atom readily forms strong, linear N···I‐X (X = O, Cl) halogen bonds with the positive *σ*‐hole on the iodine atom of the ICl or COOI fragments. For adjacent 3,5‐I_2_Py‐ICl adducts, the potential for weaker, secondary C‐I···Cl halogen bonds is also evident. Notably, the ESP maps indicate a significant overall reduction in electron density across the halogenated pyridine rings, rendering their centers strongly electropositive. This feature induces a novel interlayer packing motif distinct from the π‐π stacking commonly exploited in birefringent crystal design. Specifically, this electropositive character promotes interlayer anion‐π interactions with the chloride anions (Cl^−^) from the ICl units.

### 2.2 Crystal Structure Analysis

**FIGURE 2:**
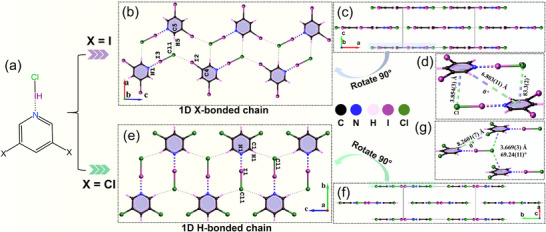
(a) Molecular structure of 3,5‐X_2_Py‐ICl; (b) 1D [3,5‐I_2_Py‐ICl] chain in **Hybrid‐XOF‐1**; (c) 3D complex framework of **Hybrid‐XOF‐1**; (d) interlayer anion‐π interactions in **Hybrid‐XOF‐1**; (e) 1D [3,5‐Cl_2_Py‐ICl] chain in **XB‐HOF‐1**; (f) 3D complex framework of **XB‐HOF‐1**; (g) interlayer anion‐π interactions in **XB‐HOF‐1**. The stipple cone lines denote strong halogen bonds, the cone lines indicate covalent bonds, and the ball lines represent weak halogen bonds or hydrogen bonds.

The parent crystals, **HOF‐1** and **HOF‐2**, are constructed from 3,4‐PDCA and 3,5‐PDCA molecules, respectively, forming extensive 3D and 2D supramolecular frameworks mediated by N‐H···O and O‐H···O hydrogen bonds (Figures S3–S6). Following halogenation, the resulting crystals, **XOF‐1**, **Hybrid‐XOF‐1**, and **XB‐HOF‐1**, exhibit a similar hierarchical assembly pattern (Figure 2; Figures S7–S15). In all three cases, the FBBs first assemble into 1D chains, which are then organized in‐plane to form quasi‐2D layers. These layers subsequently stack into a complex 3D supramolecular architecture, a process critically governed by anion‐π interactions. To quantify the strength and geometry of the key XBs, we employed the normalized contact (N_c_) parameter, defined as N_c_ = D(A‐D)/(r_A_ + r_D_) [55, 56, 57, 58, 59, 60, 61], where D(A‐D) is the measured interatomic distance and r_A_/r_D_ are the van der Waals radii of the acceptor and donor atoms.

The three halogenated structures display a similar optimization of FBB alignment. In **XOF‐1**, strong, nearly linear N···I‐O halogen bonds [D(I‐N) = 2.300(9) Å, N_c_ = 0.64, ∠N‐I‐O = 174.9(3)°] link adjacent molecules into 1D chains along the *c*‐axis (Figure S8). While the small N_c_ values confirm a strong three‐center, four‐electron interaction, the 3,5‐I_2_Py plane is nearly orthogonal to the COOI group, with a dihedral angle of 87.8(6)°. This perpendicular arrangement is detrimental to maximizing birefringence due to the cancellation of anisotropy. In **Hybrid‐XOF‐1**, the 1D chains along the *c*‐axis are co‐stabilized by two types of XBs: a strong N···I‐Cl bond [D(I3‐N1) = 2.284(12) Å, N_c_ = 0.63, ∠N‐I‐Cl = 178.2(3)°] and a weaker, secondary C‐I···Cl interaction [D(I2‐Cl1) = 3.309(3) Å, N_c_ = 0.86] (Figure 2b; Figures S11a and S12). This synergistic bonding forces all 3,5‐I_2_Py planes into a perfectly coplanar arrangement; however, the highly anisotropic N···I‐Cl units are tilted by approximately 29° relative to the chain direction. In contrast, the intra‐layer assembly in **XB‐HOF‐1** achieves a highly ordered alignment. Here, the structure is dominated by strong, perfectly linear N···I‐Cl halogen bonds [D(I‐N) = 2.39(2) Å, N_c_ = 0.66, ∠N‐I‐Cl = 180°], with weak C‐H···Cl hydrogen bonds, rather than secondary XBs, linking the FBBs into 1D chains along the *c*‐axis (Figure 2e; Figures S11b and S14). Critically, a pair of mirror‐symmetric C‐H···Cl hydrogen bonds flanking the N···I‐Cl unit locks these highly anisotropic linear moieties into a parallel orientation along the *b*‐axis. This optimal arrangement achieves both perfect coplanarity of the 3,5‐Cl_2_Py planes and parallel alignment of the N···I‐Cl bonds, resulting in a 2D layered structure with enhanced in‐plane optical anisotropy. This structure exemplifies the synergy of our strategy: the halogenated π‐system provides large intrinsic anisotropy, while the N···I‐Cl halogen bonds lock these units into the ideal coplanar and collinear geometry.

In these 3D architectures, anion‐π interactions replace π‐π stacking as the primary interlayer binding mode (Figure 2; Figure S9). This is particularly evident in **Hybrid‐XOF‐1** and **XB‐HOF‐1**, where the inter‐layer centroid‐to‐centroid distances of the pyridine rings are 6.803(11) Å and 8.2601(7) Å, respectively, effectively precluding any significant π‐π stacking (Figure 2d,g). Instead, the chloride anion (Cl^−^) of the ICl unit engages in a crucial anion‐π interaction with the electropositive face of a neighboring pyridine ring. The measured distances between the Cl^−^ and the ring centroid are 3.854(3) Å in **Hybrid‐XOF‐1** and 3.669(3) Å in **XB‐HOF‐1** (Figure 2d,g). Notably, even in **XOF‐1**, an analogous anion‐π‐like interaction between the polarized terminal iodine atom of the COOI group and an adjacent aromatic ring [D(I‐centroid) = 3.872(1) Å] is structurally more significant than the weak, parallel‐displaced *π–π* interaction present [D(centroid‐centroid) = 5.064(9) Å] (Figure S9).

**FIGURE 3:**
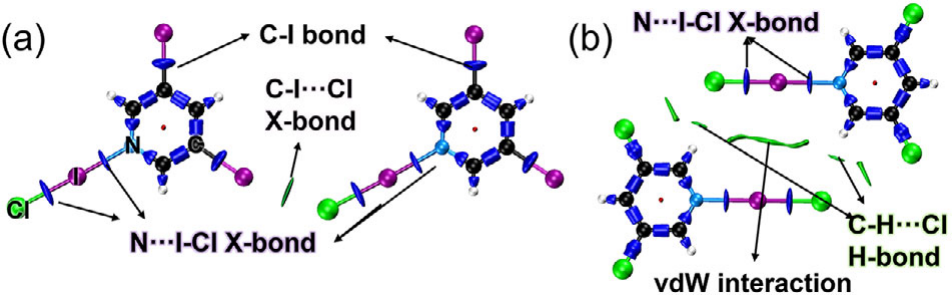
Interaction Region Indicator (IRI) analysis between adjacent FBBs in (a) **Hybrid‐XOF‐1** and (b) **XB‐HOF‐1**. Deep blue regions (sign(λ_2_)ρ ≈ ‐1) represent strong attractive interactions (such as covalent and strong halogen bonds), green regions (sign(λ_2_)ρ ≈ 0) indicate weak van der Waals interactions, and red regions (sign(λ_2_)ρ ≈ +1) denote strong repulsive forces.

To further visualize the covalent bonds and non‐covalent interactions within **Hybrid‐XOF‐1** and **XB‐HOF‐1**, we performed an Interaction Region Indicator (IRI) analysis (Figure 3) [62, 63]. For the N···I‐Cl halogen bond in both structures, the IRI isosurface between the iodine and nitrogen atoms is large and colored deep blue, comparable in signature to the C‐I covalent bonds on the pyridine ring and the I‐Cl bond itself. This signature confirms the strong, near‐covalent character of the N···I‐Cl interaction, consistent with a three‐center, four‐electron model. In contrast, the secondary C‐I···Cl interaction in **Hybrid‐XOF‐1** is visualized as a smaller, green isosurface between the iodine and chlorine atoms, indicating a much weaker interaction, closer to van der Waals forces. For **XB‐HOF‐1**, the IRI analysis highlights that adjacent FBBs are linked primarily by weak C‐H···Cl hydrogen bonds and van der Waals forces between iodine atoms, corroborating the structural data. These findings were further validated by Independent Gradient Model based on Hirshfeld partitioning (IGMH) analysis, which not only confirmed the strong halogen bonding between the 3,5‐X_2_Py and ICl fragments but also visualized the crucial anion‐π interactions between the chloride anion of the ICl unit and the pyridine rings of adjacent layers (Figure S16). Additionally, Hirshfeld surface analysis substantiated the presence of weak intra‐layer C‐I···Cl halogen bonds and C‐H···Cl hydrogen bonds, as well as the dominant inter‐layer anion‐π interactions (Figure S17).

### 2.3 Characterization of Properties

**FIGURE 4:**
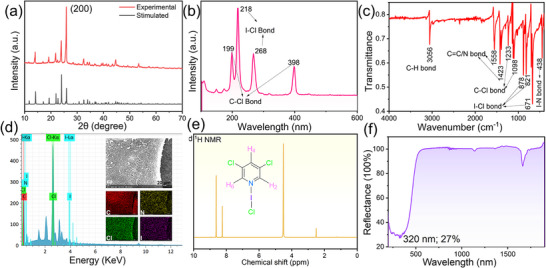
(a) PXRD pattern; (b) Raman spectrum; (c) IR spectrum; (d) EDS analysis, SEM mapping and its elemental distribution maps; (e) ^1^H NMR spectrum; (f) UV–vis diffuse reflectance spectrum of **XB‐HOF‐1**.

The phase purity of bulk crystalline samples was confirmed by powder X‐ray diffraction (PXRD), and the experimental patterns matched well with the simulated results from single‐crystal data (Figure 4a). The molecular structures and the existence elements of the FBBs were further verified by Raman spectroscopy, infrared (IR) spectroscopy, field‐emission scanning electron microscope (FESEM) analyses, mass spectrometry and ^1^H NMR spectra (Figures S19–S23 and Tables S9 and S10). In the Raman spectra, characteristic vibrations of halogen substituents were clearly observed: for **XOF‐1**, weak peaks at 131 and 150 cm^−^^1^ correspond to C‐I bending, while a strong peak at 278 cm^−^^1^ arises from C‐I stretching; for **Hybrid‐XOF‐1**, characteristic C‐I vibrations appear at 172 and 284 cm^−^^1^, along with a distinct I‐Cl peak at 267 cm^−^^1^; and for **XB‐HOF‐1**, C‐Cl peaks are observed at 199 and 398 cm^−^^1^, with an additional I‐Cl feature at 218 cm^−^^1^ (Figure 4b). The IR spectra confirmed the presence of pyridine C‐H stretching vibrations (∼3050–3060 cm^−^^1^) and skeletal vibrations (∼1500–1560 cm^−^^1^) in all FBBs. Notably, characteristic bands associated with N···I halogen bonding were detected at 675 and 671 cm^−^^1^ in **Hybrid‐XOF‐1** and **XB‐HOF‐1**, respectively (Figure 4c). Thermogravimetric analysis (TGA) revealed good thermal stability for all three compounds, with decomposition temperatures above 100°C (Figure S24). Moreover, UV–vis diffuse reflectance spectra showed absorption edges at 304, 316, and 320 nm for **XOF‐1**, **Hybrid‐XOF‐1**, and **XB‐HOF‐1**, respectively, confirming their transparency across the UV and visible regions (Figure 4f; Figure S25).

### 2.4 Experimental and Theoretical Birefringence

**FIGURE 5:**
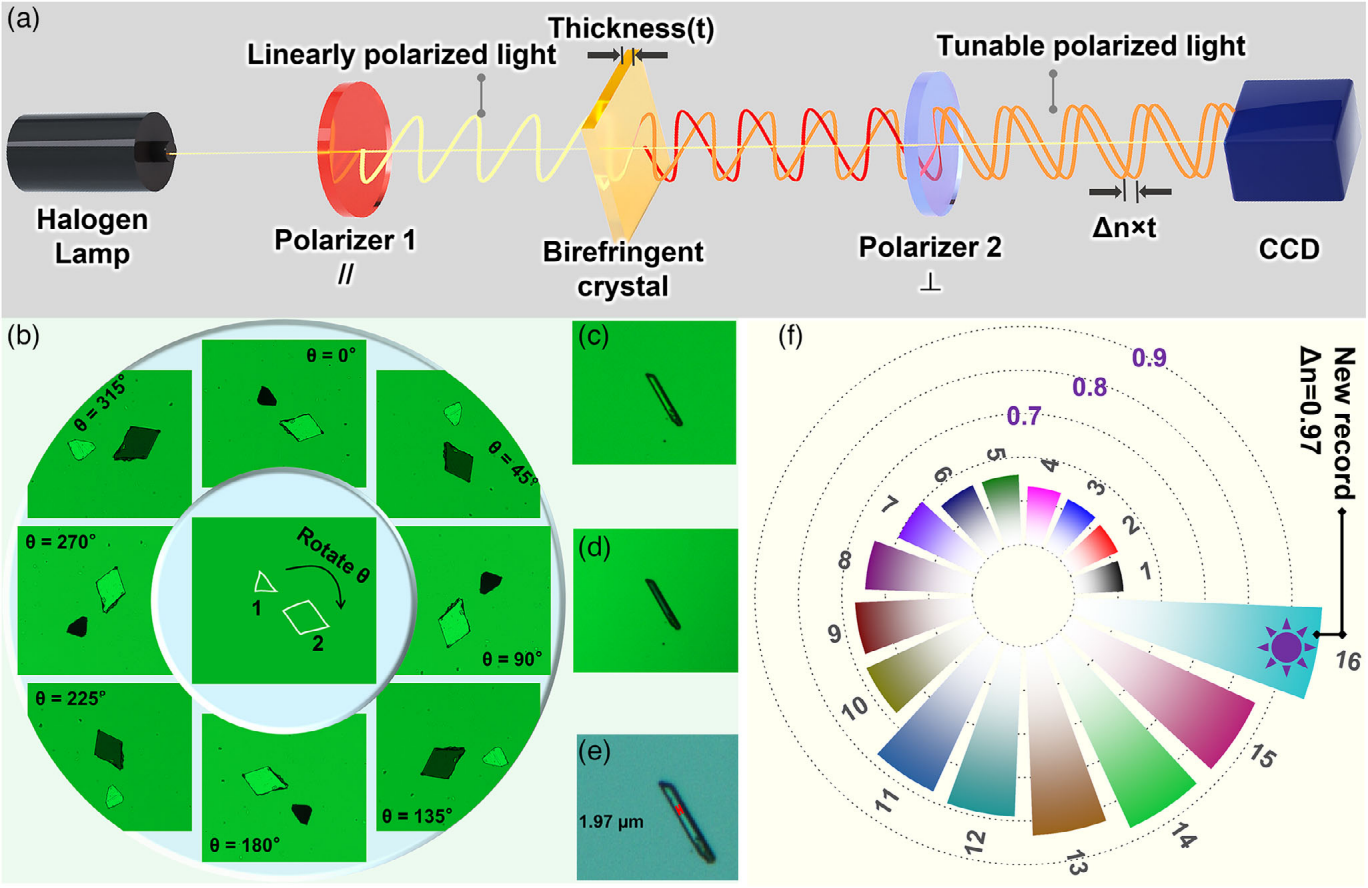
(a) Optical path diagram for birefringence measurement under polarized light microscopy; (b) brightness variation of two independent birefringent crystals with different crystal faces; (c) crystal sample at maximum brightness; (d) completely extinguished sample after compensation; (e) thickness of the measured sample; (f) Comparison of experimental birefringence values (Δn at 546 nm) for high‐performance pyridine‐based compounds (Δn > 0.5), all consistently measured using the compensator method. Bar lengths correspond to the Δn values. The numbered compounds (1–16) are detailed in Table S1. Notably, compounds 10, 15, and 16 are **XOF‐1**, **Hybrid‐XOF‐1**, **XB‐HOF‐1**, respectively. The purple numbers (e.g., 0.9, 0.7) indicate the Δn value for their corresponding circular reference lines.

The birefringence (Δ*n*) of the single crystals was determined experimentally using the Berek compensator method on a polarized light microscope, as illustrated in Figure 5a and Figure S2 [64, 65]. The principle relies on measuring the optical path difference, or retardation (*R*), induced by a crystal of known thickness (*t*). When placed between crossed polarizers, the crystals exhibit distinct brightness changes upon rotation, a hallmark of birefringence (Figure 5b, shown for **XB‐HOF‐1**). The measurement involves rotating the crystal to its brightest position (Figure 5c), inserting and adjusting the Berek compensator to achieve complete extinction (Figure 5d) to quantify *R*, and precisely measuring the crystal thickness *t* (Figure 5e). The birefringence is then calculated using the formula Δ*n* = *R* / *t*. At a wavelength of 546 nm, this method revealed exceptionally high experimental Δ*n* values for all three halogenated compounds (Figure 4; Figures S26–S28 and Table S11). **XOF‐1** exhibited a large Δ*n* of 0.68 (*R* = 1139.5 nm, *t* = 1.67 µm), which increased to a remarkable 0.87 for **Hybrid‐XOF‐1** (*R* = 2063.7 nm, *t* = 2.36 µm). Most strikingly, **XB‐HOF‐1** achieved a record‐shattering Δ*n* of 0.97 (*R* = 1902.64 nm, *t* = 1.97 µm). This exceptional birefringence is a direct consequence of its ideal crystal packing, wherein the coplanar Cl_2_Py rings and the collinear N···I‐Cl halogen bond arrays are perfectly aligned, maximizing the collective optical anisotropy.

To contextualize this outstanding performance, we systematically benchmarked our materials against both commercially available and state‐of‐the‐art birefringent crystals (Figure 5f; Table S1). The Δ*n* values of all three target compounds significantly exceed those of widely used commercial materials such as CaCO_3_ (Δ*n* = 0.172), TiO_2_ (Δ*n* = 0.256), α‐BBO (Δ*n* = 0.12), and YVO_4_ (Δ*n* = 0.20). [6, 7, 8, 9, 10] They also outperform recently reported inorganic promising materials, including Mo(H_2_O)Te_2_O_7_ (0.528 @ 546 nm) [66], Hg_4_(Te_2_O_5_)(SO_4_) (0.542 @ 546 nm) [67], and BaTeSeS_2_ (0.55 @ 550 nm) [68]. The superiority of our molecular engineering strategy is further highlighted by comparison with the parent compounds **HOF‐1** (Δ*n* = 0.12) and **HOF‐2** (Δ*n* = 0.49), demonstrating a dramatic enhancement upon halogenation (Figure S26). Within the field of Py‐ring‐containing materials, the Δn of 0.87 for **Hybrid‐XOF‐1** rivals the highest reported values, including Na_2_(4‐HPyH)_2_(PTS)·H_2_O (Δn = 0.811 @ 546 nm) [35] and (C_10_H_6_NO_2_)_2_SbF (0.87 @ 546 nm) [36]. The birefringence of 0.97 for **XB‐HOF‐1** is also comparable to or larger than recently reported UV‐transparent crystals, such as K_2_HC_9_N_13_·3H_2_O (0.87 @ 550 nm) [30], HOFBC‐1‐250 (0.800 @ 546 nm) [5], BaSbBS_4_ (0.95 @ 550 nm) [69], CsICl_2_ (0.994 @ 546 nm) [6] and Li_3_(C_9_N_13_)·6H_2_O (Δn = 1.031) [16]. Moreover, compared with previously reported solution‐processable crystals containing both π‐conjugated planes and linear units, including C_3_H_8_N_6_I_6_·3H_2_O (Δn_max_ = 2.8, 1.68 eV, UV–vis inactive) [1], [H‐4AP][IBr_2_] (0.836 @ 546 nm, 2.11 eV) [41], and INA (0.788 @ 550 nm, 3.06 eV) [43], **XB‐HOF‐1** (0.97 @ 546 nm, 2.62 eV, λ_cutoff_ = 320 nm) shows more balanced performance and represents the highest optical anisotropy among UV–vis active crystals.

**FIGURE 6:**
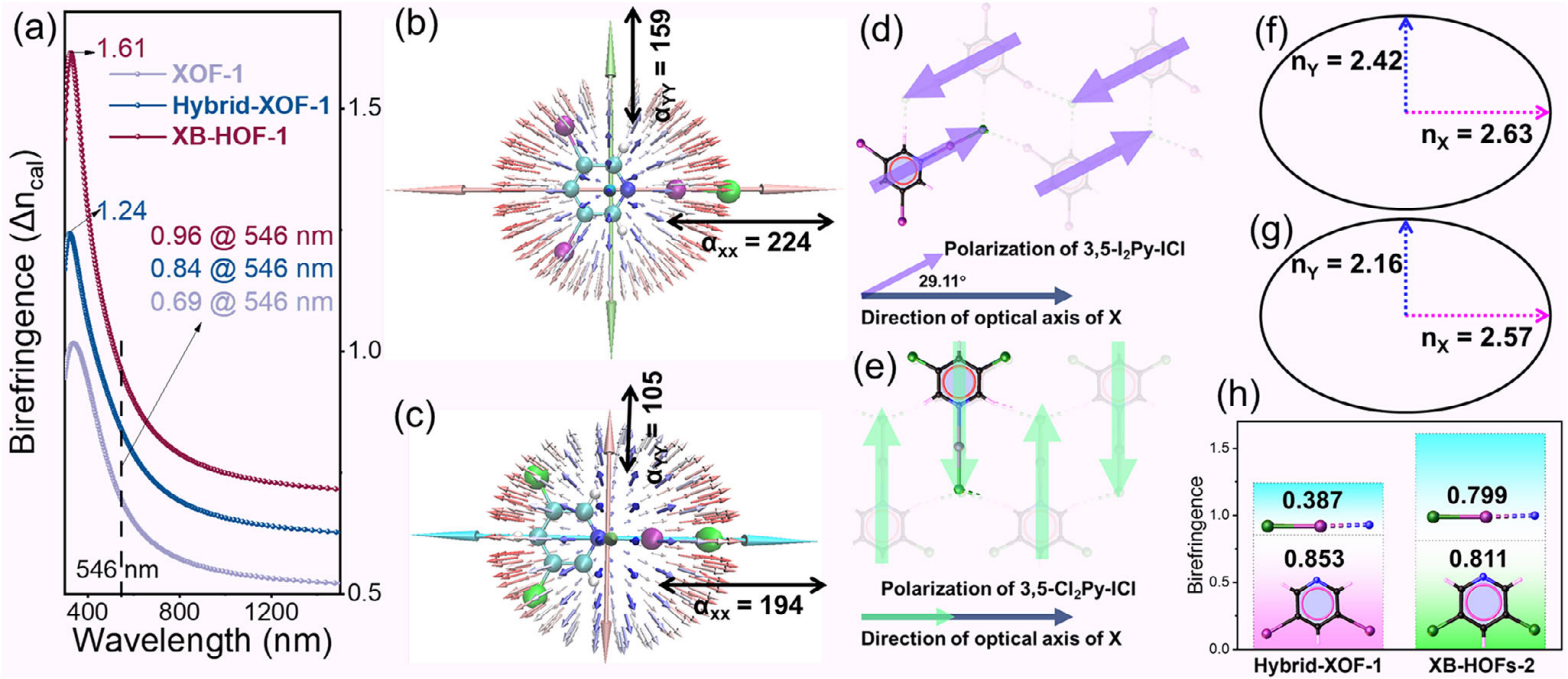
(a) Calculated birefringence values of **XOF‐1**, **Hybrid‐XOF‐1**, and **XB‐HOF‐1**; (b,c) unit sphere representations of polarizability for 3,5‐I_2_Py‐ICl (b) and 3,5‐Cl_2_Py‐ICl (c) under a static electric field; (d,e) comparison of 2D molecular packing for 3,5‐I_2_Py‐ICl (d) and 3,5‐Cl_2_Py‐ICl (e) in **Hybrid‐XOF‐1** and **XB‐HOF‐1**, with purple and light green arrows indicating the orientation of molecular polarization vectors relative to the principal optical axis (X‐axis); (f,g) schematic illustrations of in‐plane anisotropy in **Hybrid‐XOF‐1** (f) and **XB‐HOF‐1** (g); (h) Δα‐cutting analysis of birefringence for **Hybrid‐XOF‐1** and **XB‐HOF‐1**.

To corroborate these experimental findings and overcome the inherent limitation of the compensator method, which measures Δ*n* but not the absolute refractive indices, we performed first‐principles theoretical calculations. The refractive indices were derived from the frequency‐dependent complex dielectric function, *ε*(*ω*) = *ε*_1_(*ω*) + i*ε*_2_(*ω*), using the relation *n* ^2^(*ω*) = *ε*_1_(*ω*) in the transparent region. As the compounds crystallize in monoclinic and orthorhombic systems, they are biaxial, necessitating a coordinate transformation from the crystallographic axes (*a*, *b*, *c*) to the principal optical axes (*X*, *Y*, *Z*) defined by *n*_X_ > *n*_Y_ > *n*_Z_ (Table S12). The calculated refractive indices for all three compounds exhibit strong out‐of‐plane anisotropy (*n*_X_ > *n*_Y_ >> *n*_Z_) across the visible to near‐infrared spectrum, a direct result of the highly coplanar arrangement of the π‐conjugated FBBs (Figures S29 and S30). Notably, the difference between *n*_X_ and *n*_Y_ is significantly larger in **XB‐HOF‐1**, which we attribute to the enhanced in‐plane anisotropy arising from the perfectly parallel alignment of the N···I‐Cl bonds. Using the relation Δ*n* = *n*_X_ – *n*_Z_, the calculated birefringence values at 546 nm are 0.69, 0.84, and 0.96 for **XOF‐1**, **Hybrid‐XOF‐1**, and **XB‐HOF‐1**, respectively (Figure 6a). This excellent agreement between theory and experiment validates the reliability of our measurements. Furthermore, these theoretical values far exceed those calculated for the parent compounds **HOF‐1** (0.12) and **HOF‐2** (0.50), theoretically confirming the efficacy of our design strategy (Figure S29). The calculations also predict that **XB‐HOF‐1** possesses a maximum theoretical birefringence approaching an extraordinary value of 1.61, highlighting its immense potential.

### 2.5 Structure‐Property Relationship

The systematic evolution from the parent material **HOF‐1** to the champion crystal **XB‐HOF‐1**, culminating in a colossal birefringence of 0.97, provides a direct and powerful validation of our halogen‐bond coupled halogenated‐π‐conjugation strategy. This strategy operates through a dual mechanism: first, the halogenated‐π‐conjugation enhances the intrinsic polarizability anisotropy (Δα) while suppressing the out‐of‐plane refractive index (n_Z_); second, the halogen‐bond coupling (N···I‐Cl) acts as a directional anchor, rigidly locking these anisotropic units into a collinear and coplanar arrangement to maximize the macroscopic birefringence. First, the transition from **HOF‐1** (Δ*n*_exp_ = 0.68 for its derivative, **XOF‐1**) to the **HOF‐2** series highlights the critical importance of π‐conjugated unit alignment. In **XOF‐1**, the π‐conjugated moieties (3,5‐I_2_Py and COOI) are arranged nearly orthogonally, which severely restricts the constructive superposition of their polarizabilities and thus limits the overall birefringence. In contrast, the FBBs in **HOF‐2**, **Hybrid‐XOF‐1**, and **XB‐HOF‐1** are engineered to be coplanar or nearly coplanar. This parallel arrangement is a foundational requirement for achieving large birefringence. Secondly, within this coplanar framework, the dramatic performance leap from **HOF‐2** (Δ*n*_exp_ = 0.49) to **Hybrid‐XOF‐1** (0.87) and **XB‐HOF‐1** (0.97) is driven by a sharp increase in the FBBs' polarizability anisotropy. This was achieved by replacing the carboxylate groups of the 3,5‐PDCA precursor with linear, highly polarizable N···I‐Cl halogen‐bonding linkers. This targeted molecular engineering directly enhances the optical anisotropy of the building blocks themselves.

A notable finding, however, is that **XB‐HOF‐1** exhibits superior birefringence despite its FBB (3,4‐Cl_2_Py‐ICl) possessing a lower calculated polarizability anisotropy than that of **Hybrid‐XOF‐1** (3,5‐I_2_Py‐ICl) (144 vs. 172 a.u.). This counterintuitive result underscores that optimal crystal packing can be even more critical than maximizing individual molecular properties. The origin of this difference lies in the directional alignment of the FBBs' principal polarizability tensor, which for both molecules is oriented along the linear N···I‐Cl halogen bond axis (Figure 6b,c; Figure S31). In **Hybrid‐XOF‐1**, competing secondary C‐I···Cl interactions between adjacent FBBs induce a corrugated chain packing, which forces a ∼29° misalignment between the highly anisotropic N···I‐Cl vectors and the principal optical axis (*X*‐axis) of the crystal (Figure 6d). This angular deviation prevents the full projection of the molecular anisotropy onto the macroscopic optical axes, thereby diminishing the bulk birefringence. In stark contrast, the packing in **XB‐HOF‐1**, governed by weaker C‐H···Cl hydrogen bonds, enforces a perfectly linear and coplanar chain arrangement. This ideal geometry ensures excellent parallelism, aligning every N···I‐Cl vector collinearly with the *X*‐axis (Figure 6e). This optimal orientation leads to a significantly larger in‐plane refractive index difference (*n*_X_ – *n*_Y_) in **XB‐HOF‐1** (0.41) compared to **Hybrid‐XOF‐1** (0.21) (Figure 6f,g).

To quantify this effect, we decomposed the total theoretical birefringence into contributions from the planar π‐conjugated unit (e.g., 3,5‐Cl_2_Py) and the linear N···I‐Cl unit (Figure 6h; Table S13). The analysis reveals that the perfect alignment in **XB‐HOF‐1** causes the contribution from the N···I‐Cl unit to more than double compared to **Hybrid‐XOF‐1** (0.799 vs. 0.387). Remarkably, this packing‐induced enhancement also elevates the contribution of the 3,5‐Cl_2_Py ring to a level comparable to that of the 3,5‐I_2_Py ring, despite the latter's intrinsically higher polarizability. This funding highlights a key principle of our halogen‐bond coupled halogenated‐π‐conjugation strategy: the halogen‐bond coupling element must be optimized to align the highly anisotropic N···I‐X vectors along a single crystallographic direction.

### 2.6 Electronic Structure Analysis

To elucidate the electronic origins of the observed optical properties, we performed first‐principles calculations. Theoretical predictions indicate that all three compounds are indirect bandgap semiconductors, with calculated bandgaps of 2.728 eV, 2.532 eV, and 2.686 eV for **XOF‐1**, **Hybrid‐XOF‐1**, and **XB‐HOF‐1**, respectively (Figure S32). Analysis of the partial density of states (PDOS) reveals the orbital contributions at the band edges. Taking the champion **XB‐HOF‐1** as a representative example, its valence band maximum (VBM) is primarily derived from C‐2p and I‐5p orbitals, with minor contributions from Cl‐3p and N‐2p orbitals. The conduction band minimum (CBM), in turn, is composed of I‐5p, Cl‐3p, and C‐2p orbitals (Figure S33). This demonstrates a strong hybridization between the *p*‐orbitals of the halogen atoms (I, Cl) and the π‐conjugated framework (C, N), resulting in a highly delocalized electronic structure that is conducive to large optical anisotropy.

**FIGURE 7:**
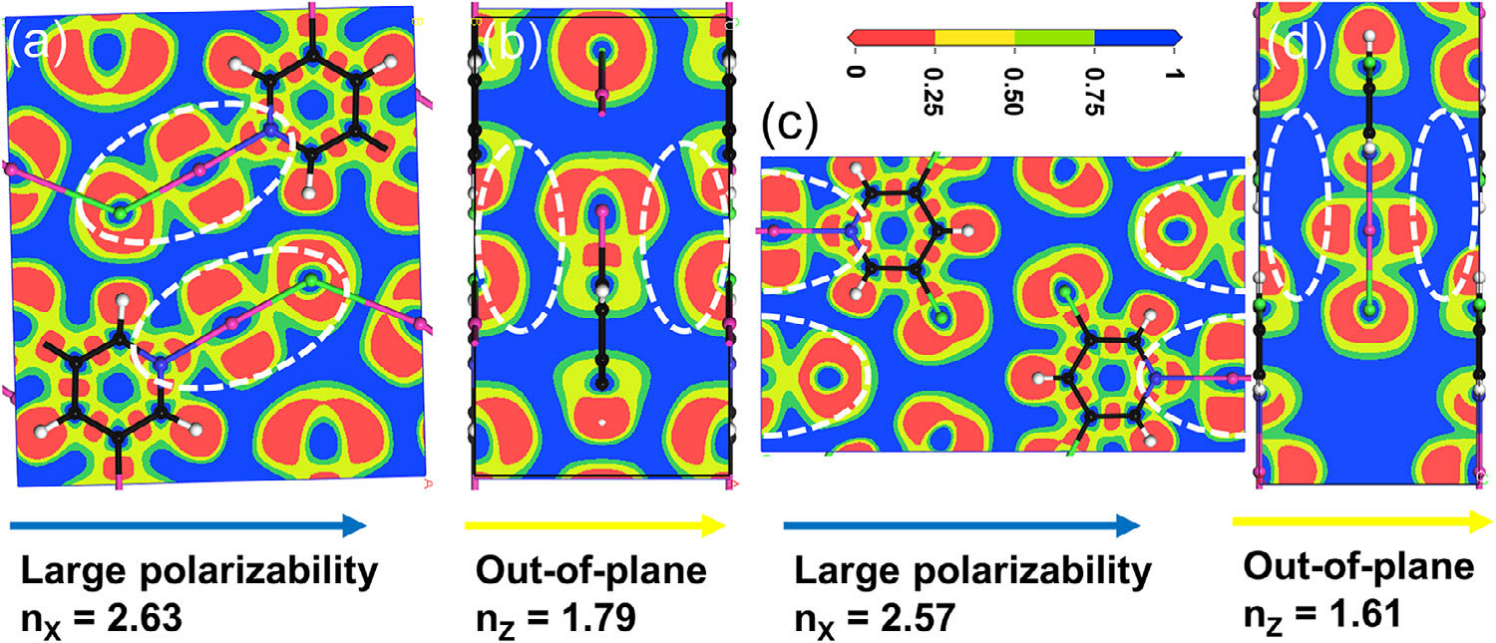
(a, c) In‐plane ELF maps of **Hybrid‐XOF‐1** and **XB‐HOF‐1** showing the orientation of N···I‐Cl bond electron density; (b, d) out‐of‐plane ELF maps of **Hybrid‐XOF‐1** and **XB‐HOF‐1** revealing the interlayer electron density.

The electron localization function (ELF) provides further insight into this anisotropy at the electronic level. For both **Hybrid‐XOF‐1** and **XB‐HOF‐1**, the ELF maps reveal a clear difference between the in‐plane (Figure 7a,c) and out‐of‐plane (Figure 7b,d) directions. Strong, continuous electron localization is observed along the covalent and halogen bonds within the 2D layers, whereas localization is significantly weaker between the layers. This pronounced electronic anisotropy is the fundamental origin of their giant birefringence. Importantly, the ELF analysis also explains an apparent contradiction: why does **XB‐HOF‐1** exhibit higher birefringence than **Hybrid‐XOF‐1**, even though its principal refractive index (*n*_X_ = 2.57) is lower than that of **Hybrid‐XOF‐1** (*n*_X_ = 2.63)? The answer lies in the out‐of‐plane polarizability, quantified by *n*_Z_. The diffuse electron cloud of the iodine substituents in **Hybrid‐XOF‐1** leads to a substantially higher out‐of‐plane refractive index (*n*_Z_ = 1.79) compared to the chlorine‐substituted **XB‐HOF‐1** (*n*_Z_ = 1.61). This is visually corroborated by the lower electron density in the interlayer region of **XB‐HOF‐1** (Figure 7b and 6d). Therefore, the strategy of replacing the ring iodine atoms with less polarizable chlorine atoms achieves a dual benefit for maximizing birefringence (Δ*n* = *n*_X_ – *n*_Z_): it not only enables the optimal in‐plane packing (coplanar and collinear) but also simultaneously minimizes the undesirable out‐of‐plane polarizability. This synergistic optimization is a key factor behind the outstanding birefringence of **XB‐HOF‐1**.

## 3 Conclusion

In conclusion, we have developed a halogen‐bond coupled halogenated‐π‐conjugation strategy that leverages the synergy between strong halogen bonds and weak secondary interactions to create a new class of birefringent crystals with exceptional performance. By systematically evolving from simple hydrogen‐bonded precursors to complex halogen‐bonded frameworks, we achieved a giant experimental birefringence of 0.97 in the champion crystal, **XB‐HOF‐1**, a value validated by first‐principles calculations. Our combined experimental and theoretical analysis reveals a profound structure‐property principle: optimal crystal packing is critical and can even override the intrinsic anisotropy of the molecular building blocks. The outstanding performance of **XB‐HOF‐1** stems directly from a packing motif that enforces near‐perfect coplanar and collinear alignment of the anisotropic units. This work not only delivers a material with outstanding birefringence but also illuminates a transferable design pathway for rationally engineering the next generation of ultrahigh‐performance optical crystals. This synergistic strategy is expected to be applicable to other heterocyclic and anionic systems for developing high‐performance optical materials.

## 4 Methods

CCDC 2476068 for **XOF‐1**, 2476069 for **Hybrid‐XOF‐1**, and 2476070 for **XB‐HOF‐1**, contains the supplementary crystallographic data for this paper. These data can be obtained free of charge from The Cambridge Crystallographic Data Centre via www.ccdc.cam.ac.uk/data_request/cif.

The authors have cited additional references of Methods within the Supporting Information [70, 71, 72, 73, 74, 75, 76, 77, 78, 79, 80, 81, 82].

## Funding

National Natural Science Foundation of China (Nos. 22573015 and 22373014).

## Conflicts of Interest

The authors declare no conflicts of interest.

## Supporting information




**Supporting File**: advs73877‐sup‐0001‐SuppMat.docx.

**Supporting File**: advs73877‐sup‐0002‐Data.zip.

## Data Availability

The data that support the findings of this study are available from the corresponding author upon reasonable request.
