# Supplementary material for: Halogen‐Bond Coupled Halogenated‐π‐Conjugation Enables Giant Birefringence in Hydrogen‐Bonded Organic Frameworks
Source: Adv Sci (Weinh). 2026 Jan 14;13(17):e00006. doi: 10.1002/advs.202600006 (PMC13042645; doi:10.1002/advs.202600006)
Supplement: Supplementary file 1 — Supporting File: advs73877‐sup‐0001‐SuppMat.docx. [file ADVS-13-e00006-s001.docx]

**Halogen-Bond Coupled Halogenated-π-Conjugation Enables Giant Birefringence in Hydrogen-Bonded Organic Frameworks**

*Miao-Bin Xu, Yun-Xia Hu, Ming-Chang Wang, Jia-Jia Li, Jia-Min Lian, Jin Chen,* and Ke-Zhao Du**

Fujian Provincial Key Laboratory of Advanced Materials Oriented Chemical Engineering, College of Chemistry and Material Science, Fujian Normal University, Fuzhou 350007, China
E-mail: cj2015@fjnu.edu.cn; duke@fjnu.edu.cn

Jin Chen, and Ke-Zhao Du
State Key Laboratory of Structural Chemistry, Fujian Institute of Research on the Structure of Matter, Chinese Academy of Sciences, Fuzhou 350002, P. R. China

Miao-Bin Xu and Yun-Xia Hu contributed equally to this work.

Table of contents

| **Methods** | S4 |
| --- | --- |
| **Figure S1**. Schematic illustration of the synthetic routes | S4 |
| **Figure S2.** An illustration of the compensator method. | S7 |
| **Table S1.** Comparison of the experimental birefringence | S10 |
| **Table S2.** Crystal data and structure refinement for **HOF-1** and **HOF-2** | S11 |
| **Table S3.** Crystal data and structure refinement for **XOF-1**, **Hybrid-XOF-1**, and **XB-HOF-1** | S12 |
| **Table S4.** Fractional Atomic Coordinates and Equivalent Isotropic Displacement Parameters | S13 |
| **Table S5.** Bond Lengths for title compounds | S14 |
| **Table S6.** Bond Angles for title compounds | S16 |
| **Table S7.** Hydrogen Bond in **XB-HOF-1** | S17 |
| **Table S8.** Theoretical and experimental results of elemental analysis for title compounds | S17 |
| **Table S9.** Raman spectra of title compounds | S17 |
| **Table S10.** The assignments of the infrared absorption peaks for title compounds | S18 |
| **Table S11.** Birefringence test of title compounds | S18 |
| **Table S12.** The transformations from principal dielectric axes to principal optical axes | S18 |
| **Table S13.** The birefringence of units in **Hybrid-XOF-1** and **XB-HOF-1** | S18 |
| **Figure S3**. Asymmetric units in (a) **HOF-1** (3,4-PDCA) and (b) **HOF-2** (3,5-PDCA) | S20 |
| **Figure S4**. Hydrogen-bonding motifs in (a) **HOF-1** and (b) **HOF-2**. | S20 |
| **Figure S5.** (a) 1D [3,4-PDCA] hydrogen-bonded chain and (b) 3D framework of **HOF-1**. | S21 |
| **Figure S6**. (a) Two-dimensional [3,5-PDCA] hydrogen-bonded layer, (b) overall structure of **HOF-2** viewed along the *c* axis, and (c) interlayer anion-π interactions. | S21 |
| **Figure S7.** The asymmetric units of (a) 3,5-I₂Py-COOI, (b) 3,5-I₂Py-ICl, and (c) 3,5-Cl₂Py-ICl | S22 |
| **Figure S8.** Packing diagram of 3,5-I₂Py-COOI viewed along the crystallographic *a*-axis | S22 |
| **Figure S9.** The 3D supramolecular architecture of **XOF-1**, viewed along the *c*-axis | S23 |
| **Figure S10**. The 3D supramolecular architecture of **XOF-1**, viewed along the *b*-axis. | S23 |
| **Figure S11**. Halogen/hydrogen-bonding interactions in (a) **Hybrid-XOF-1** and (b) **XB-HOF-1**. | S24 |
| **Figure S13**. Perspective view of the 3D **Hybrid-XOF-1** framework along the crystallographic *a* axis | S25 |
| **Figure S14.** Packing diagram of 3,5-Cl₂Py-ICl viewed along the crystallographic *a*-axis in **XB-HOF-1**, illustrating its 2D pseudo-layered structure | S26 |
| **Figure S15**. The 3D **XB-HOF-1** framework along the crystallographic *a* axis | S26 |
| **Figure S16**. IGMH isosurface plots | S26 |
| **Figure S17.** Hirshfeld surfaces mapped with d_norm_ | S27 |
| **Figure S18.** Experimental and simulated PXRD patterns | S27 |
| **Figure S19.** ¹H NMR spectra | S28 |
| **Figure S20.** Field-emission scanning electron microscope (FESEM) analyses | S29 |
| **Figure S21.** High-resolution mass spectrometry analysis | S30 |
| **Figure S22.** Raman spectra | S31 |
| **Figure S23.** IR spectra | S31 |
| **Figure S24.**TG and DTA curves | S32 |
| **Figure S25.** UV-Vis-IR spectra and optical bandgaps | S32 |
| **Figure S26.** Birefringence characterization of the parent molecules, **HOF-1** and **HOF-2**. | S33 |
| **Figure S27.** Optical characterization of a single crystal of **XOF-1** for birefringence measurement. | S33 |
| **Figure S28.** Optical characterization of a single crystal of **Hybrid-XOF-1** for birefringence measurement. | S34 |
| **Figure S29.** The calculated refractive index and birefringence of **HOF-1** (a) and **HOF-2** (b) | S34 |
| **Figure S30.** The calculated refractive index and birefringence of **XOF-1** (a), **Hybrid-XOF-1** (b) and **XB-HOF-1** (c) | S35 |
| **Figure S31.** Unit sphere representation of polarizability for 3,5-I_2_Py-ICl (a) and 3,4-I_2_Py-ICl (b) in **Hybrid-XOF-1** and **XB-HOF-1**, respectively, under a static electric field | S35 |
| **Figure S32.** Calculated bandgaps of **XOF-1** (a), **Hybrid-XOF-1** (b) and **XB-HOF-1** (c) | S36 |
| **Figure S33.** The PDOS of **XOF-1** (a), **Hybrid-XOF-1** (b) and **XB-HOF-1** (c) | S36 |
| **Reference** | S37 |

**Methods**

**Materials and Synthesis**

**Materials**. 3,4-Pyridinedicarboxylic acid (C₇H₅NO₄, 98%), 3,5-pyridinedicarboxylic acid (C₇H₅NO₄, 98%+), iodine(V) oxide (I₂O₅, 99.0%), selenium dioxide (SeO_2_, 99.0%), hydrochloric acid (HCl, 36–38%), nitric acid (HNO₃, 65–68%), and sulfuric acid (H₂SO₄, 96–98%) were purchased from Adamas-beta and used as received without further purification.

**Synthesis of HOF-1 (3,4-PDCA)**. A mixture of 3,4-Pyridinedicarboxylic acid (3,4-PDCA), 167.1 mg, 1.0 mmol), SeO_2_ (111.0 mg, 1.0 mmol), HNO₃ (0.1 mL), and H₂O (1.0 mL) was sealed in a 23 mL Teflon-lined stainless-steel autoclave. The autoclave was heated to 110 °C for 2 days and then slowly cooled to 30 °C at a rate of 3.3 °C/h. This procedure yielded colorless block-shaped crystals of **HOF-1**. Yield: ~90% (based on 3,4-PDCA).

**Synthesis of HOF-2 (3,5-PDCA)**. A mixture of 3,5-Pyridinedicarboxylic acid (3,5-PDCA), 167.1 mg, 1.0 mmol), SeO_2_ (133.2 mg, 1.0 mmol), HNO₃ (0.1 mL), and H₂O (1.0 mL) was sealed in a 23 mL Teflon-lined stainless-steel autoclave. The autoclave was heated to 110 °C for 2 days and then slowly cooled to 30 °C at a rate of 3.3 °C/h. This procedure yielded colorless block-shaped crystals of **HOF-2**. Yield: ~80% (based on 3,5-PDCA).

**XOF-1、Hybrid-XOF-1**和**XB-HOF-1** were prepared via a one-pot method using **HOF-1** and **HOF-2** as the parent materials (after grinding) (Figure S1).


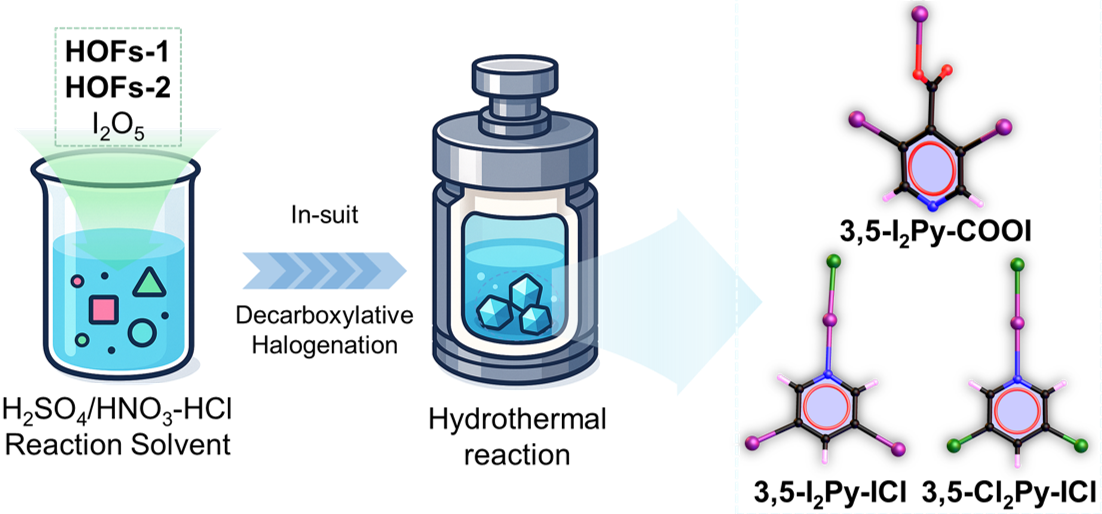


**Figure S1**. Schematic illustration of the synthetic routes for (a) XOF-1, (b) **Hybrid-XOF-1**, and (c) **XB-HOF-1**.

**Synthesis of XOF-1 (3,5-I_2_Py-COOI)**. A mixture of **HOF-1** (3,4-PDCA) (167.1 mg, 1.0 mmol), I₂O₅ (333.8 mg, 1.0 mmol), H₂SO₄ (0.5 mL), and H₂O (1.0 mL) was sealed in a 23 mL Teflon-lined stainless-steel autoclave. The autoclave was heated to 130 °C for 3 days and then slowly cooled to 30 °C at a rate of 1.9 °C/h. This procedure yielded colorless rod-shaped crystals of 1. Yield: ~80% (based on 3,4-pyridinedicarboxylic acid).

**Synthesis of Hybrid-XOF-1 (3,5-I₂Py-ICl)**. A mixture of **HOF-2** (3,5-PDCA) (83.6 mg, 0.5 mmol), I₂O₅ (166.9 mg, 0.5 mmol), HCl (0.3 mL), HNO₃ (0.3 mL), and H₂O (1.0 mL) was sealed in a 23 mL Teflon-lined stainless-steel autoclave. The autoclave was heated to 110 °C for 3 days and then slowly cooled to 30 °C at a rate of 1.1 °C/h. This procedure yielded colorless rod-shaped crystals of 2. Yield: ~75% (based on 3,5-pyridinedicarboxylic acid).

**Synthesis of XB-HOF-1 (3,5-Cl₂Py-ICl)**. A mixture of **HOF-2** (3,5-PDCA) (83.6 mg, 0.5 mmol), I₂O₅ (100.0 mg, 0.3 mmol), HCl (0.5 mL), HNO₃ (0.5 mL), and H₂O (3.0 mL) was sealed in a 23 mL Teflon-lined stainless-steel autoclave. The autoclave was heated to 110 °C for 3 days and then slowly cooled to 30 °C at a rate of 1.1 °C/h. This procedure yielded colorless flaky crystals of 3. Yield: ~75% (based on I₂O₅ as the limiting reagent).

**Single crystal structure determination**

**Crystal Selection and Mounting.** Single crystals of **HOF-1**, **HOF-2**, **XOF-1**, **Hybrid-XOF-1** and **XB-HOF-1** suitable for X-ray diffraction were selected under a polarizing microscope. Each crystal was mounted on a MiTeGen MicroMount™ using Paratone-N oil and immediately transferred to the goniometer.

**Data Collection**. X-ray intensity data were collected on a Rigaku XtaLAB Synergy-DW dual-wavelength CCD diffractometer. Data for **HOF-1**, **HOF-2**, and **XOF-1** were collected at 293(2) K using graphite-monochromated Cu Kα radiation (λ = 1.54184 Å). Data for **Hybrid-XOF-1** and **XB-HOF-1** were collected at 296(2) K and 293(2) K, respectively, using graphite-monochromated Mo Kα radiation (λ = 0.71073 Å). The ω-scan method was employed for data collection.

**Data Processing**. The collected diffraction data were processed using the CrysAlisPro software suite (Version 1.171.40.68a). The processing included integration of the diffraction spots, Lorentz and polarization corrections, and a multi-scan absorption correction based on the crystal faces.^1^

**Structure Solution and Refinement.** The structures of **HOF-1**, **HOF-2**, **XOF-1**, **Hybrid-XOF-1** and **XB-HOF-1** were solved by direct methods using SHELXT and refined by full-matrix least-squares on F² using the SHELXL-2014 program within the Olex2 graphical user interface.^2^ All non-hydrogen atoms were refined with anisotropic thermal parameters. Hydrogen atoms attached to carbon were placed in geometrically calculated positions and refined using a riding model with C–H = 0.93 Å (aromatic) and Uᵢₛₒ(H) = 1.2Uₑ₁(C). The final structures were checked for additional or missing symmetry elements using the PLATON program, and none were found.^3^ Final refinement statistics, including R-factors, goodness-of-fit (GooF), and residual electron densities, are provided in the crystallographic tables. Detailed crystallographic data and structure refinement parameters are summarized in Tables S2–S7.

**Crystallographic Data.** Crystallographic data have been deposited with the Cambridge Crystallographic Data Centre (CCDC). Deposition Numbers: CCDC 2476068 for **XOF-1**, 2476069 for **Hybrid-XOF-1**, and 2476070 for **XB-HOF-1**. These data can be obtained free of charge from The Cambridge Crystallographic Data Centre via www.ccdc.cam.ac.uk/data_request/cif.

**Powder X-ray diffraction**

Powder X-ray diffraction patterns were collected at room temperature (298 K) on a Rigaku Ultima IV diffractometer using graphite-monochromated Cu Kα radiation (λ = 1.54184 Å). The samples were finely ground and mounted on a flat, zero-background sample holder. Data were recorded over a 2θ range of 10° to 50° with a step size of 0.02° and a scan speed of 2°/min.

**Thermal analysis**

Thermogravimetric analysis (TGA) and differential thermal analysis (DTA) were performed simultaneously using a Rigaku TG-DTA 8121 thermal analyzer. A sample mass of approximately 5 mg was placed in an alumina crucible and heated from 30 °C to 800 °C at a constant rate of 10 °C/min. The measurements were conducted under a dynamic argon atmosphere with a flow rate of 50 mL/min.

**Optical measurements**

**Infrared (IR) Spectroscopy**. Fourier-transform infrared (FT-IR) spectra were recorded on a Thermo Fisher Scientific Nicolet 5700 spectrometer. The samples were prepared as KBr pellets, and spectra were collected over the range of 4000–400 cm⁻¹ with a resolution of 4 cm⁻¹. Each spectrum was an average of 32 scans.

**UV-Vis Diffuse Reflectance Spectroscopy**. Ultraviolet-visible (UV-vis) diffuse reflectance spectra were measured using a PerkinElmer Lambda 750 spectrophotometer equipped with an integrating sphere accessory. BaSO₄ was used as a 100% reflectance standard. Data were collected from 200 to 2000 nm. The raw reflectance data (R) were converted into absorption data (F(R)) using the Kubelka-Munk equation: F(R) = (1−R)² / (2R).^4^

**Raman Spectroscopy**. Raman spectra were recorded using a LabRAM Odyssey Raman spectrometer (HORIBA Scientific, Japan) with a 532 nm linearly polarized argon laser as the excitation source. The laser beam was focused onto the sample through a 100× objective lens, resulting in a spot size of approximately 10 μm in diameter. The laser power was maintained at a low level (e.g., < 1 mW) to prevent sample degradation.

**Birefringence**


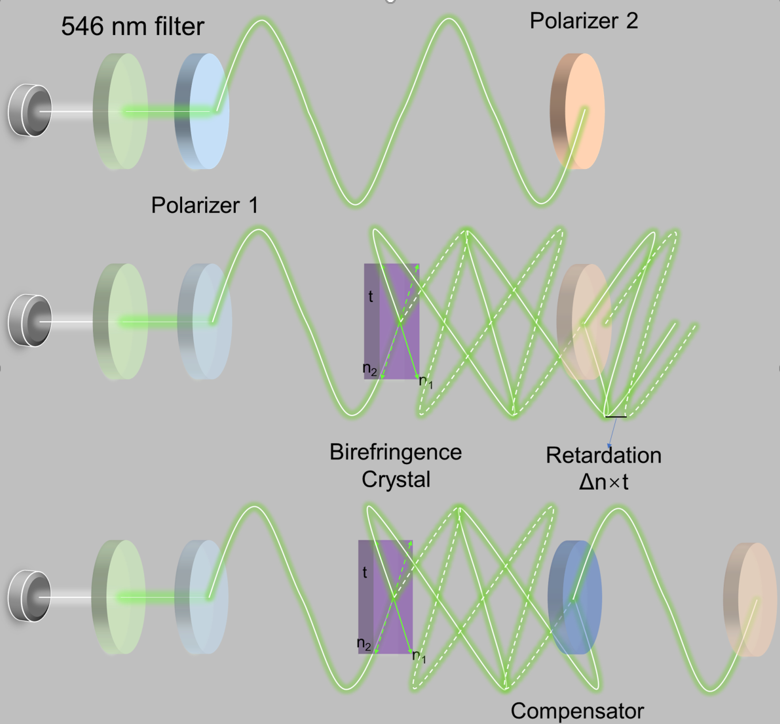


**Figure S2.** An illustration of the compensator method. (a) Typical crossed polarizer setup. Light is polarized by the polarizer 1 and does not pass through the polarizer 2 (analyzer). (b) A birefringent crystal of thickness t is placed between the polarizers, creating elliptically polarized light with a retardation of ∆n×t. The light now has some component in the direction of the analyzer. (c) A compensator is placed between the crystal and the analyzer and tilted until complete compensation of the retarded light back to being linearly polarized in the direction of the analyzer.

Birefringence (Δn) measurements were conducted on a Nikon LV1000 polarizing microscope equipped with a Berek compensator.^5^ The principle of the measurement is illustrated in Figure S1. In a standard crossed-polarizer setup, linearly polarized light from the first polarizer (polarizer) is blocked by the second, orthogonally oriented polarizer (analyzer), resulting in a dark field of view (Figure S2a).

When a birefringent crystal is placed between the polarizers, it splits the incoming linearly polarized light into two orthogonal waves (fast and slow) that travel at different speeds. This difference in speed, governed by the material's different refractive indices (n_e_ and n_o_), induces a phase difference, or retardation (R = Δn·*t*), where Δn = |n_e_ - n_o_| and *t* is the crystal thickness. Upon exiting the crystal, these out-of-phase waves recombine to form elliptically polarized light (Figure S1b). This elliptically polarized light now has a component that can pass through the analyzer, causing the crystal to appear bright. When rotating the crystal stage, extinction (the crystal appears black) occurs every 90° when one of the crystal's principal refractive index directions aligns with the polarizer's axis. Maximum brightness is observed when the principal axes are at a 45° angle to the polarizer.

To quantify the retardation, a compensator—a calibrated crystal of known optical properties—is inserted into the light path between the sample and the analyzer (Figure S2c). The compensator is oriented to introduce a retardation that is equal in magnitude but opposite in sign to that of the sample. By tilting the compensator, its effective retardation is adjusted until it perfectly cancels the retardation from the sample. At this point of "complete compensation," the light becomes linearly polarized again and is fully blocked by the analyzer, causing the crystal to reach maximum extinction. The retardation value (R) is read directly from the calibrated compensator.

For each compound, a high-quality single crystal with smooth faces was selected. The retardation (R) was measured at a wavelength of 546 nm. Subsequently, the crystal was carefully oriented on its side using double-sided tape, and its thickness (t) was measured using OLYMPUS Stream software.^6^ The birefringence (Δn) was calculated using the formula Δn = R / *t*. The measurement process for each compound is shown in Figures S24-S26.

**Computational Method**

The electronic structures and optical properties of the compounds were computed using the plane-wave pseudopotential method within the framework of density functional theory (DFT), as implemented in the total energy code CASTEP.^7, 8^ For the exchange-correlation functional, we selected the Perdew-Burke-Ernzerhof (PBE) formula within the generalized gradient approximation (GGA).^9^ The interactions between the ionic cores and the valence electrons were modeled using norm-conserving pseudopotentials.^10^ The following orbital electrons were treated as valence electrons: C-2s^2^2p^2^, N-2s^2^2p^3^, O-2s^2^2p^4^, Cl-3s^2^3p^5^, I-5s^2^5p^5^, H-1s. The basis set was constructed with plane waves up to a cutoff energy of 750 eV. For both compounds, the self-consistent field (SCF) and optical-property calculations utilized a k-point separation of 0.04 Å^-1^ for the numerical integration over the Brillouin zone. The k-point samplings employed were 3 × 2 × 3 for XOF-1, 2 × 4 × 2 for Hybrid-XOF-1, 4 × 2 × 3 for **XB-HOF-1**.^7, 8^

To investigate the polarizability anisotropy and electronic structure of selected birefringent units, a systematic computational approach was undertaken using the Gaussian 09 software suite.^11^ The Hybrid B3LYP functional was employed at the 6-31G(d,p) level of theory for these calculations. Subsequent analysis of the computational results was performed using the Multiwfn 3.8 software package.^12^ The polarizability anisotropy was quantified based on the static polarizability values. To explore electrostatic potential (ESP), systematic calculations were implemented via the Gaussian 09 package at the B3LYP level of exchange functional of the DFT calculations.^11^ All the structures were calculated using the LanL2DZ basis set. Subsequent analysis of the computational results was performed using the Multiwfn 3.8 software package.^12^ The VMD was used to plot color-filled iso-surface graphs to visualize the molecular electrostatic potential (MESP). To explore independent gradient model based on Hirshfeld partition (IGMH),^13^ systematic calculations were performed using the B3LYP exchange functional level in density functional theory (DFT) calculations with the Gaussian 09 software package. All structures were calculated using the LanL2DZ basis set.^11^ Color IGMH maps were drawn using VMD software.

To obtain the linear optical properties, the complex dielectric function ε(ω) = ε_1_(ω) + iε_2_(ω) has been determined in the random phase approximation from the PBE wavefunctions. The imaginary part of the dielectric function due to direct inter-band transitions is given by the expression,

where *Ω, ω, u, ν* and *c* are the unit-cell volume, photon frequencies, the vector defining the polarization of the incident electric field, valence and conduction bands, respectively. The real part of the dielectric function is obtained from *ε_2_* by a Kramers-Kronig transformation,

$$\varepsilon_{1}(\omega)=1+(\frac{2}{\pi})\int_{0}^{+\infty} d\omega'\frac{\omega'^{2}\varepsilon_{2}(\omega)}{\omega'^{2}-\omega^{2}}$$

**Table S1.** Comparison of the experimental birefringence of the title compounds with previously reported state-of-the-art pyridine-based crystals exhibiting Δn_exp_ > 0.5.

| Compounds | Number | Birefringence | Ref. |
| --- | --- | --- | --- |
| **HOF-1** |  | 0.12 @ 546 nm | **This work** |
| **HOF-2** |  | 0.49 @ 546 nm |  |
| (C_5_H_5_NO)(Sb_2_OF_4_) | 1 | 0.513 @ 546 nm | 14 |
| Li-4HP_2_ | 2 | 0.522 @ 546 nm | 15 |
| (C_5_H_6_NO)^+^(HC_2_O_4_)^-^ | 3 | 0.526 @ 546 nm | 16 |
| [HPyClB(OH)_2_]·(NO_3_) | 4 | 0.533 @ 546 nm | 17 |
| (C_4_N_3_H_6_)^+^(NO_3_)^-^ | 5 | 0.56 @ 546 nm | 16 |
| (C_5_H_6_NO)^+^(NO_3_)^-^ | 6 | 0.562 @ 546 nm | 16 |
| [Be_2_(μ-OH)_2_(C_7_H_3_NO_4_)(H_2_O)]·H_2_O | 7 | 0.6 @ 546 nm | 18 |
| [H-4AP][ICl_2_] | 8 | 0.647 @ 546 nm | 19 |
| (C_5_H_6.16_N_2_Cl_0.84_)(IO_2_Cl_2_) | 9 | 0.67 @ 546 nm | 20 |
| **XOF-1** | 10 | 0.68 @ 546 nm | **This work** |
| INA | 11 | 0.778 @ 546 nm | 21 |
| (C_12_H_8_N_2_)SbF_3_ | 12 | 0.79 @ 546 nm | 22 |
| [H-4AP][IBr₂] | 13 | 0.833 @ 546 nm | 19 |
| (C_10_H_6_NO_2_)_2_SbF | 14 | 0.87 @ 546 nm | 23 |
| **Hybrid-XOF-1** | 15 | 0.87 @ 546 nm | **This work** |
| **XB-HOF-1** | 16 | 0.97 @ 546 nm |  |

The number 1-16 related the compounds in Figure 5f.

**Table S2.** Crystal data and structure refinement for **HOF-1** and **HOF-2**.

| Compounds | **HOF-1** | **HOF-2** |
| --- | --- | --- |
| Empirical formula | C_7_H_5_NO_4_ | C_7_H_5_NO_4_ |
| Formula weight | 167.12 | 167.12 |
| Temperature/K | 293(2) | 293(2) |
| Crystal system | orthorhombic | monoclinic |
| Space group | *P*2_1_2_1_2_1_ | *P*2_1_/*c* |
| a/Å | 5.2814(4) | 9.7069(8) |
| b/Å | 11.1717(7) | 11.1619(7) |
| c/Å | 11.2437(9) | 6.5954(7) |
| α/° | 90 | 90 |
| β/° | 90 | 107.804(10) |
| γ/° | 90 | 90 |
| Volume/Å^3^ | 663.40(8) | 680.37(11) |
| Z | 4 | 4 |
| ρ_calc_ g/cm^3^ | 1.673 | 1.632 |
| μ/mm^‑1^ | 1.219 | 1.189 |
| F(000) | 344.0 | 344.0 |
| Radiation | CuKα (λ = 1.54184) | CuKα (λ = 1.54184) |
| Goodness-of-fit on *F*^2^ | 1.067 | 1.103 |
| Final R indexes [I>=2σ (I)] | R_1_ = 0.0638, wR_2_ = 0.1679 | R_1_ = 0.0568, wR_2_ = 0.1458 |
| Final R indexes [all data] | R_1_ = 0.0704, wR_2_ = 0.1733 | R_1_ = 0.0737, wR_2_ = 0.1589 |

**Table S3.** Crystal data and structure refinement for **XOF-1**, **Hybrid-XOF-1**, and **XB-HOF-1**.

| Compounds | **XOF-1** | **Hybrid-XOF-1** | **XB-HOF-1** |
| --- | --- | --- | --- |
| Empirical formula | C_6_H_2_I_3_NO_2_ | C_5_H_3_ClI_3_N | C_5_H_3_Cl_3_IN |
| Formula weight | 500.79 | 493.23 | 310.33 |
| Temperature/K | 293(2) | 296.15 | 293(2) |
| Crystal system | monoclinic | monoclinic | orthorhombic |
| Space group | *P*2_1_/n | *C*2/m | Cmcm |
| a/Å | 7.8721(6) | 12.830(3) | 6.8624(9) |
| b/Å | 14.2084(9) | 7.1170(15) | 15.0274(14) |
| c/Å | 9.4218(6) | 11.404(2) | 9.1706(9) |
| α/° | 90 | 90 | 90 |
| β/° | 103.631(6) | 91.537(18) | 90 |
| γ/° | 90 | 90 | 90 |
| Volume/Å^3^ | 1024.15(12) | 1040.9(4) | 945.71(18) |
| Z | 4 | 4 | 4 |
| ρ_calc_ g/cm^3^ | 3.248 | 3.147 | 2.180 |
| μ/mm^‑1^ | 71.639 | 9.202 | 4.163 |
| F(000) | 880.0 | 864.0 | 576.0 |
| Radiation | CuKα (λ = 1.54184) | MoKα (λ = 0.71073) | MoKα (λ = 0.71073) |
| Goodness-of-fit on *F*^2^ | 1.069 | 1.084 | 1.059 |
| Final R indexes [I>=2σ (I)] | R_1_ = 0.0631, wR_2_ = 0.1684 | R_1_ = 0.0601, wR_2_ = 0.1443 | R_1_ = 0.0961, wR_2_ = 0.2119 |
| Final R indexes [all data] | R_1_ = 0.0723, wR_2_ = 0.1769 | R_1_ = 0.0820, wR_2_ = 0.1654 | R_1_ = 0.0998, wR_2_ = 0.2140 |

**Table S4.** Fractional Atomic Coordinates (×10^4^) and Equivalent Isotropic Displacement Parameters (Å^2^×10^3^) for title compounds.

| **HOF-1 (3,4-PDCA)** | | | | | | | | | |
| --- | --- | --- | --- | --- | --- | --- | --- | --- | --- |
| Atom | | x | | y | | z | | U(eq) | |
| O4 | | 7099(6) | | 6046(3) | | 911(3) | | 34.1(8) | |
| O2 | | 2437(7) | | 4304(3) | | 4307(3) | | 37.4(8) | |
| O3 | | 5252(8) | | 4467(3) | | 1753(3) | | 42.8(9) | |
| O1 | | 5847(7) | | 5450(3) | | 4553(4) | | 47.9(11) | |
| N1 | | 744(7) | | 7983(3) | | 1973(4) | | 31.6(9) | |
| C6 | | 5443(8) | | 5533(3) | | 1594(4) | | 25.6(8) | |
| C7 | | 3752(8) | | 5206(3) | | 4118(3) | | 23.5(8) | |
| C5 | | 2625(9) | | 7322(3) | | 1524(4) | | 28.8(9) | |
| C3 | | 2688(7) | | 6174(3) | | 3319(3) | | 21.8(8) | |
| C4 | | 3611(7) | | 6384(3) | | 2172(4) | | 23.3(8) | |
| C2 | | 770(9) | | 6899(4) | | 3757(4) | | 31.0(9) | |
| C1 | | -196(9) | | 7795(4) | | 3054(4) | | 31.8(9) | |
| **HOF-2 (3,5-PDCA)** | | | | | | | | | |
| Atom | | x | | y | | z | | U(eq) | |
| O12 | | 3890.8(18) | | 4712.0(15) | | 7651(3) | | 45.8(5) | |
| O11 | | 4171(2) | | 6701.8(15) | | 7710(4) | | 57.1(6) | |
| O9 | | 8457.9(19) | | 2271.2(14) | | 7454(3) | | 50.8(5) | |
| O8 | | 10379.1(18) | | 3420.6(15) | | 7811(4) | | 56.5(6) | |
| N6 | | 8183(2) | | 6524.9(17) | | 7127(4) | | 43.5(5) | |
| C2 | | 8205(2) | | 4410(2) | | 7473(4) | | 37.1(5) | |
| C4 | | 6121(2) | | 5544.5(19) | | 7520(4) | | 36.1(5) | |
| C3 | | 6818(2) | | 4452.6(19) | | 7627(4) | | 37.2(5) | |
| C5 | | 6842(3) | | 6572(2) | | 7272(4) | | 40.6(6) | |
| C7 | | 9041(2) | | 3263(2) | | 7586(4) | | 40.3(6) | |
| C10 | | 4608(3) | | 5590.4(19) | | 7639(4) | | 37.4(5) | |
| C1 | | 8860(3) | | 5478(2) | | 7233(4) | | 42.3(6) | |
| **XOF-1 (3,5-I_2_Py-COOI)** | | | | | | | | |  |
| Atom | x | | y | | z | | U(eq) | |  |
| I1 | 8045.8(10) | | 4136.1(5) | | 325.9(6) | | 36.3(3) | |  |
| I2 | 7284.9(13) | | 6306.3(7) | | 4655.4(9) | | 52.5(3) | |  |
| I3 | 7755.7(15) | | 2029.2(7) | | 4566.9(10) | | 57.3(3) | |  |
| O1 | 8417(12) | | 4178(6) | | 2682(9) | | 41.7(19) | |  |
| N1 | 7912(13) | | 4127(7) | | 7861(10) | | 34(2) | |  |
| C6 | 7712(16) | | 3312(8) | | 5649(11) | | 34(2) | |  |
| C1 | 7094(16) | | 4175(9) | | 3191(11) | | 38(3) | |  |
| C3 | 7494(16) | | 4982(9) | | 5619(10) | | 35(2) | |  |
| C2 | 7509(15) | | 4173(8) | | 4867(11) | | 32(2) | |  |
| C5 | 7908(17) | | 3314(9) | | 7135(11) | | 38(3) | |  |
| O2 | 5614(19) | | 4135(10) | | 2533(15) | | 77(4) | |  |
| C4 | 7706(16) | | 4946(10) | | 7153(11) | | 39(3) | |  |
| **Hybrid-XOF-1 (3,5-I_2_Py-ICl)** | | | | | | | | |  |
| Atom | x | | y | | z | | U(eq) | |  |
| I1 | 5774.7(10) | | 5000 | | 7519.7(12) | | 71.8(4) | |  |
| I2 | 1682.8(10) | | 5000 | | 10005.3(9) | | 62.3(4) | |  |
| I3 | 1676.1(8) | | 5000 | | 4642.4(8) | | 46.9(4) | |  |
| Cl1 | 772(4) | | 5000 | | 2693(3) | | 61.8(10) | |  |
| N1 | 2558(10) | | 5000 | | 6410(10) | | 46(3) | |  |
| C1 | 3615(12) | | 5000 | | 6487(12) | | 46(3) | |  |
| C2 | 4145(12) | | 5000 | | 7506(13) | | 47(4) | |  |
| C3 | 3599(12) | | 5000 | | 8526(13) | | 47(3) | |  |
| C4 | 2578(16) | | 5000 | | 8497(13) | | 57(4) | |  |
| C5 | 2038(13) | | 5000 | | 7414(12) | | 49(3) | |  |
| **XB-HOF-1 (3,5-Cl_2_Py-ICl)** | | | | | | | | |  |
| Atom | x | | y | | z | | U(eq) | |  |
| I1 | 5000 | | 4750.6(13) | | 7500 | | 78.2(9) | |  |
| Cl1 | 5000 | | 6400(5) | | 7500 | | 79(2) | |  |
| Cl2 | 5000 | | 1278(5) | | 4554(9) | | 125(3) | |  |
| N1 | 5000 | | 3163(14) | | 7500 | | 56(5) | |  |
| C1 | 5000 | | 2731(15) | | 6230(20) | | 71(6) | |  |
| C2 | 5000 | | 1811(12) | | 6200(30) | | 70(6) | |  |
| C3 | 5000 | | 1350(20) | | 7500 | | 90(11) | |  |

**Table S5.** Bond Lengths for title compounds.

| **HOF-1** | | | | | | | | | | | | | |
| --- | --- | --- | --- | --- | --- | --- | --- | --- | --- | --- | --- | --- | --- |
| Atom | | Atom | | Length/Å | |  | | Atom | | Atom | | Length/Å | |
| O4 | | C6 | | 1.297(5) | |  | | C6 | | C4 | | 1.504(6) | |
| O2 | | C7 | | 1.242(5) | |  | | C7 | | C3 | | 1.514(5) | |
| O3 | | C6 | | 1.208(6) | |  | | C5 | | C4 | | 1.378(6) | |
| O1 | | C7 | | 1.240(5) | |  | | C3 | | C4 | | 1.398(6) | |
| N1 | | C5 | | 1.337(6) | |  | | C3 | | C2 | | 1.387(6) | |
| N1 | | C1 | | 1.330(6) | |  | | C2 | | C1 | | 1.374(7) | |
| **HOF-2** | | | | | | | | | | | | | |
| Atom | | Atom | | Length/Å | |  | | Atom | | Atom | | Length/Å | |
| O12 | | C10 | | 1.204(3) | |  | | C2 | | C3 | | 1.382(3) | |
| O11 | | C10 | | 1.317(3) | |  | | C2 | | C7 | | 1.506(3) | |
| O9 | | C7 | | 1.234(3) | |  | | C2 | | C1 | | 1.383(3) | |
| O8 | | C7 | | 1.273(3) | |  | | C4 | | C3 | | 1.385(3) | |
| N6 | | C5 | | 1.336(3) | |  | | C4 | | C5 | | 1.379(3) | |
| N6 | | C1 | | 1.332(3) | |  | | C4 | | C10 | | 1.496(3) | |
| **XOF-1** | | | | | | | | | | | | |  |
| Atom | Atom | | Length/Å | |  | | Atom | | Atom | | Length/Å | |  |
| I1 | O1 | | 2.171(8) | |  | | C6 | | C2 | | 1.417(16) | |  |
| I1 | N1^1^ | | 2.300(9) | |  | | C6 | | C5 | | 1.372(14) | |  |
| I2 | C3 | | 2.078(12) | |  | | C1 | | C2 | | 1.535(13) | |  |
| I3 | C6 | | 2.093(12) | |  | | C1 | | O2 | | 1.185(18) | |  |
| O1 | C1 | | 1.246(16) | |  | | C3 | | C2 | | 1.353(17) | |  |
| N1 | C5 | | 1.342(16) | |  | | C3 | | C4 | | 1.417(13) | |  |
| N1 | C4 | | 1.331(15) | |  | |  | |  | |  | |  |
| ^1^+X,+Y,-1+Z | | | | | | | | | | | | |  |
| **Hybrid-XOF-1** | | | | | | | | | | | | |  |
| Atom | Atom | | Length/Å | |  | | Atom | | Atom | | Length/Å | |  |
| I1 | C2 | | 2.091(16) | |  | | N1 | | C5 | | 1.340(18) | |  |
| I2 | Cl1^1^ | | 3.309(4) | |  | | C1 | | C2 | | 1.33(2) | |  |
| I2 | C4 | | 2.094(15) | |  | | C2 | | C3 | | 1.373(18) | |  |
| I3 | Cl1 | | 2.480(4) | |  | | C3 | | C4 | | 1.31(3) | |  |
| I3 | N1 | | 2.285(12) | |  | | C4 | | C5 | | 1.40(2) | |  |
| N1 | C1 | | 1.36(2) | |  | |  | |  | |  | |  |
| ^1^+X,+Y,1+Z | | | | | | | | | | | | |  |
| **XB-HOF-1** | | | | | | | | | | | | |  |
| Atom | Atom | | Length/Å | |  | | Atom | | Atom | | Length/Å | |  |
| I1 | Cl^1^ | | 2.479(8) | |  | | N1 | | C1 | | 1.33(2) | |  |
| I1 | N1 | | 2.39(2) | |  | | C1 | | C2 | | 1.38(3) | |  |
| Cl2 | C2 | | 1.71(2) | |  | | C2 | | C3 | | 1.38(3) | |  |
| N1 | C11 | | 1.33(2) | |  | |  | |  | |  | |  |
| ^1^+X,+Y,3/2-Z | | | | | | | | | | | | |  |

**Table S6.** Bond Angles for title compounds.

| **HOF-1** | | | | | | | | | | | | | | | | | | | | | | | | | | | | | | | | | | | | |
| --- | --- | --- | --- | --- | --- | --- | --- | --- | --- | --- | --- | --- | --- | --- | --- | --- | --- | --- | --- | --- | --- | --- | --- | --- | --- | --- | --- | --- | --- | --- | --- | --- | --- | --- | --- | --- |
| Atom | | Atom | | | Atom | | | | | Angle/˚ | | | | | |  | | | | Atom | | | | | Atom | | | | Atom | | | | | Angle/˚ | | |
| C1 | | N1 | | | C5 | | | | | 122.3(4) | | | | | |  | | | | C4 | | | | | C3 | | | | C7 | | | | | 122.5(3) | | |
| O4 | | C6 | | | C4 | | | | | 114.2(3) | | | | | |  | | | | C2 | | | | | C3 | | | | C7 | | | | | 118.5(4) | | |
| O3 | | C6 | | | O4 | | | | | 125.4(4) | | | | | |  | | | | C2 | | | | | C3 | | | | C4 | | | | | 119.0(3) | | |
| O3 | | C6 | | | C4 | | | | | 120.3(4) | | | | | |  | | | | C5 | | | | | C4 | | | | C6 | | | | | 119.7(4) | | |
| O2 | | C7 | | | C3 | | | | | 118.2(4) | | | | | |  | | | | C5 | | | | | C4 | | | | C3 | | | | | 118.9(4) | | |
| O1 | | C7 | | | O2 | | | | | 127.5(4) | | | | | |  | | | | C3 | | | | | C4 | | | | C6 | | | | | 121.1(3) | | |
| O1 | | C7 | | | C3 | | | | | 114.1(3) | | | | | |  | | | | C1 | | | | | C2 | | | | C3 | | | | | 119.5(4) | | |
| N1 | | C5 | | | C4 | | | | | 120.1(4) | | | | | |  | | | | N1 | | | | | C1 | | | | C2 | | | | | 120.2(4) | | |
| **HOF-2** | | | | | | | | | | | | | | | | | | | | | | | | | | | | | | | | | | | | |
| Atom | | | Atom | | | | Atom | | | | Angle/˚ | | | |  | | | | | | Atom | | | | | Atom | | | | | Atom | | | | Angle/˚ | |
| C1 | | | N6 | | | | C5 | | | | 120.5(2) | | | |  | | | | | | N6 | | | | | C5 | | | | | C4 | | | | 121.1(2) | |
| C3 | | | C2 | | | | C7 | | | | 123.3(2) | | | |  | | | | | | O9 | | | | | C7 | | | | | O8 | | | | 124.2(2) | |
| C3 | | | C2 | | | | C1 | | | | 118.1(2) | | | |  | | | | | | O9 | | | | | C7 | | | | | C2 | | | | 122.0(2) | |
| C1 | | | C2 | | | | C7 | | | | 118.7(2) | | | |  | | | | | | O8 | | | | | C7 | | | | | C2 | | | | 113.8(2) | |
| C3 | | | C4 | | | | C10 | | | | 120.0(2) | | | |  | | | | | | O12 | | | | | C10 | | | | | O11 | | | | 125.0(2) | |
| C5 | | | C4 | | | | C3 | | | | 118.7(2) | | | |  | | | | | | O12 | | | | | C10 | | | | | C4 | | | | 123.5(2) | |
| C5 | | | C4 | | | | C10 | | | | 121.3(2) | | | |  | | | | | | O11 | | | | | C10 | | | | | C4 | | | | 111.50(19) | |
| C2 | | | C3 | | | | C4 | | | | 119.9(2) | | | |  | | | | | | N6 | | | | | C1 | | | | | C2 | | | | 121.7(2) | |
| **XOF-1** | | | | | | | | | | | | | | | | | | | | | | | | | | | | | | | | | | | |  |
| Atom | Atom | | | Atom | | | | | Angle/˚ | | | | |  | | | Atom | | | | | Atom | | | | | Atom | | | | | Angle/˚ | | | |  |
| O1 | I1 | | | N1^1^ | | | | | 174.9(3) | | | | |  | | | O2 | | | | | C1 | | | | | C2 | | | | | 118.9(12) | | | |  |
| C1 | O1 | | | I1 | | | | | 118.1(7) | | | | |  | | | C2 | | | | | C3 | | | | | I2 | | | | | 123.4(7) | | | |  |
| C5 | N1 | | | I1^2^ | | | | | 120.9(7) | | | | |  | | | C2 | | | | | C3 | | | | | C4 | | | | | 119.3(11) | | | |  |
| C4 | N1 | | | I1^2^ | | | | | 118.2(7) | | | | |  | | | C4 | | | | | C3 | | | | | I2 | | | | | 117.2(9) | | | |  |
| C4 | N1 | | | C5 | | | | | 120.7(10) | | | | |  | | | C6 | | | | | C2 | | | | | C1 | | | | | 120.5(10) | | | |  |
| C2 | C6 | | | I3 | | | | | 120.7(7) | | | | |  | | | C3 | | | | | C2 | | | | | C6 | | | | | 118.3(10) | | | |  |
| C5 | C6 | | | I3 | | | | | 119.2(9) | | | | |  | | | C3 | | | | | C2 | | | | | C1 | | | | | 120.7(10) | | | |  |
| C5 | C6 | | | C2 | | | | | 120.1(11) | | | | |  | | | N1 | | | | | C5 | | | | | C6 | | | | | 120.5(11) | | | |  |
| O1 | C1 | | | C2 | | | | | 113.7(10) | | | | |  | | | N1 | | | | | C4 | | | | | C3 | | | | | 121.0(11) | | | |  |
| O2 | C1 | | | O1 | | | | | 127.4(11) | | | | |  | | |  | | | | |  | | | | |  | | | | |  | | | |  |
| ^1^+X,+Y,-1+Z; ^2^+X,+Y,1+Z | | | | | | | | | | | | | | | | | | | | | | | | | | | | | | | | | | | |  |
| **Hybrid-XOF-1** | | | | | | | | | | | | | | | | | | | | | | | | | | | | | | | | | | | |  |
| Atom | | Atom | | | | Atom | | | | Angle/˚ | | |  | | | | | Atom | | | | | Atom | | | | | Atom | | | | | Angle/˚ | | |  |
| C4 | | I2 | | | | Cl11 | | | | 167.4(6) | | |  | | | | | C1 | | | | | C2 | | | | | C3 | | | | | 118.7(16) | | |  |
| N1 | | I3 | | | | Cl1 | | | | 178.2(3) | | |  | | | | | C3 | | | | | C2 | | | | | I1 | | | | | 121.8(13) | | |  |
| C1 | | N1 | | | | I3 | | | | 121.8(9) | | |  | | | | | C4 | | | | | C3 | | | | | C2 | | | | | 120.7(16) | | |  |
| C5 | | N1 | | | | I3 | | | | 120.5(11) | | |  | | | | | C3 | | | | | C4 | | | | | I2 | | | | | 123.4(13) | | |  |
| C5 | | N1 | | | | C1 | | | | 117.7(13) | | |  | | | | | C3 | | | | | C4 | | | | | C5 | | | | | 119.6(15) | | |  |
| C2 | | C1 | | | | N1 | | | | 122.8(14) | | |  | | | | | C5 | | | | | C4 | | | | | I2 | | | | | 117.0(14) | | |  |
| C1 | | C2 | | | | I1 | | | | 119.6(11) | | |  | | | | | N1 | | | | | C5 | | | | | C4 | | | | | 120.4(16) | | |  |
| ^1^+X,+Y,1+Z | | | | | | | | | | | | | | | | | | | | | | | | | | | | | | | | | | | |  |
| **XB-HOF-1** | | | | | | | | | | | | | | | | | | | | | | | | | | | | | | | | | | | |  |
| Atom | | | Atom | | | | | Atom | | | Angle/˚ |  | | | | | | | Atom | | | | | Atom | | | | | | Atom | | | | Angle/˚ | |  |
| N1 | | | I1 | | | | | Cl1 | | | 180.0 |  | | | | | | | C1 | | | | | C2 | | | | | | Cl2 | | | | 118.8(18) | |  |
| C1 | | | N1 | | | | | I1 | | | 119.1(12) |  | | | | | | | C3 | | | | | C2 | | | | | | Cl2 | | | | 121.8(16) | |  |
| C11 | | | N1 | | | | | I1 | | | 119.1(12) |  | | | | | | | C3 | | | | | C2 | | | | | | C1 | | | | 119(2) | |  |
| C11 | | | N1 | | | | | C1 | | | 122(2) |  | | | | | | | C2 | | | | | C3 | | | | | | C21 | | | | 119(3) | |  |
| N1 | | | C1 | | | | | C2 | | | 120(2) |  | | | | | | |  | | | | |  | | | | | |  | | | |  | |  |
| ^1^+X,+Y,3/2-Z | | | | | | | | | | | | | | | | | | | | | | | | | | | | | | | | | | | |  |

**Table S7.** Hydrogen Bond in **XB-HOF-1**.

| D | H | A | d(D-H)/Å | d(H-A)/Å | d(D-A)/Å | D-H-A/° |
| --- | --- | --- | --- | --- | --- | --- |
| C1 | H1 | Cl1^1^ | 0.93 | 2.75 | 3.66(2) | 166.6 |
| ^1^1-X,1-Y,-1/2+Z | | | | | | |

**Table S8.** Theoretical and experimental results of elemental analysis for title compounds.

| **Weight**  **(%)** | **XOF-1** | | **Hybrid-XOF-1** | | **XB-HOF-1** | |
| --- | --- | --- | --- | --- | --- | --- |
|  | exp | cal | exp | cal | exp | cal |
| C | 14.63 | 14.38 | 12.40 | 12.16 | 16.69 | 19.33 |
| H | 0.03 | 0.4 | 0 | 0.6 | 0.08 | 0.96 |
| N | 3.14 | 2.80 | 3.25 | 2.84 | 4.45 | 4.51 |
| **Ratio** |  |  |  |  |  |  |
| C | 6.10 | 6 | 5.10 | 5 | 4.3 | 5 |
| H | 0.2 | 3 | 0 | 3 | 0.25 | 3 |
| N | 1.12 | 1 | 1.15 | 1 | 0.98 | 1 |

**Table S9.** Raman spectra of title compounds

| Assignment (cm^-1^) | **XOF-1** | **Hybrid-XOF-1** | **XB-HOF-1** |
| --- | --- | --- | --- |
| C-O-I | 212 | / | / |
| C-I | 131, 150 ,212, 278 | 172, 284 | / |
| C-Cl | / | / | 199, 398 |
| I-Cl | / | 267 | 218, 268 |

**Table S10.** The assignments of the infrared absorption peaks for title compounds

| Assignment (cm^-1^) | **XOF-1** | **Hybrid-XOF-1** | **XB-HOF-1** |
| --- | --- | --- | --- |
| C-H | 3060 | 3052 | 3056 |
| C=O | 1668 | / | / |
| C=C/C=N | 1498, 1400 | 1531 | 1558, 1423 |
| C-I | 1092 | 1222, 1078 | / |
| I-O | 594, | / | / |
| N-I | / | 482 | 438 |
| I-Cl | / | 736, 675 | 671 |
| C-Cl | / | / | 1098 |

**Table S11.** Birefringence test of title compounds.

| Compounds | R (nm) | T (μm) | Δn (@546nm) |
| --- | --- | --- | --- |
| **HOF-1** | 1020.26 | 8.18 | 0.12 |
| **HOF-2** | 1279.57 | 2.59 | 0.49 |
| **XOF-1** | 1139.46 | 1.67 | 0.68 |
| **Hybrid-XOF-1** | 2063.67 | 2.36 | 0.87 |
| **XB-HOF-1** | 1902.64 | 1.97 | 0.97 |

**Table S12.** The transformations from principal dielectric axes to principal optical axes.

| Compounds | Order of calculated refractive index | Transformation |
| --- | --- | --- |
| **HOF-1** | *n*(c) > *n*(b) > *n*(a) | c→X, b→Y, a→Z |
| **HOF-2** | *n*(c) > *n*(a) > *n*(b) | c→X, a→Y, b→Z |
| **XOF-1** | *n*(b) > *n*(c) > *n*(a) | b→X, c→Y, a→Z |
| **Hybrid-XOF-1** | *n*(b) > *n*(c) > *n*(a) | b→X, c→Y, a→Z |
| **XB-HOF-1** | *n*(a) > *n*(b) > *n*(c) | a→X, b→Y, c→Z |

**Table S13.** The birefringence of units in **Hybrid-XOF-1** and **XB-HOF-1**.

| Units | Δα | γ/θ | Cos(γ/θ) | Z | V | Factor | Contribution  (100%) | Δ*n*_cut_ |
| --- | --- | --- | --- | --- | --- | --- | --- | --- |
| 3,5-I_2_Py | 113 | 0 | 1 | 4 | 1040.9 | 0.434 | 0.688 | 0.853 |
| N-I-Cl | 59 | 29.11 | 0.87 | 4 | 1040.9 | 0.197 | 0.312 | 0.387 |
| 3,5-Cl_2_Py | 64 | 0 | 1 | 4 | 945.71 | 0.271 | 0.504 | 0.811 |
| N-I-Cl | 63 | 0 | 1 | 4 | 945.71 | 0.266 | 0.496 | 0.799 |

**Δα**: The polarizability anisotropy of the specified functional unit (i.e., the planar π-conjugated ring or the linear N–I–Cl segment). **γ**: The angle between the plane of the π-conjugated unit (3,5-I₂Py or 3,4-Cl₂Py) and the crystallographic XY plane. Theoretically, a smaller γ angle signifies a greater difference between the in-plane and out-of-plane polarizability distributions, which enhances the overall optical anisotropy. **θ**: The angle between the axis of the linear N–I–Cl unit and the principal optical X-axis. A smaller θ angle indicates a more effective projection of the unit's high polarizability onto the axis of maximum refractive index, thus maximizing its contribution to birefringence. **Z**: The number of formula units per unit cell. **V**: The volume of the unit cell (Å³). **Factor**: An orientation-weighted polarizability anisotropy density, calculated using the formula: Factor = Δα × cos(γ or θ) × Z / V. This metric provides a semi-quantitative estimate of a unit's effective contribution to the crystal's overall anisotropy.

**Contribution**: The ratio of the "Factor" values for the two distinct functional units (planar π-conjugated unit vs. linear N–I–Cl unit), which normalizes their relative impact. Δn_cut_: The decomposed birefringence contribution from each unit. It is calculated by multiplying the material's maximum theoretical birefringence (Δn_max_) by the "Contribution " for that unit. This value represents the apportioned contribution of each structural component to the total birefringence.


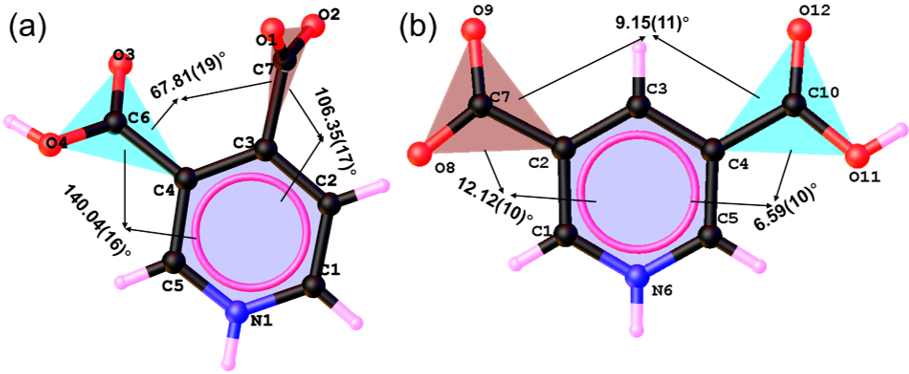


**Figure S3**. Asymmetric units in (a) **HOF-1** (3,4-PDCA) and (b) **HOF-2** (3,5-PDCA). All C, N, O, and H atoms occupy general positions. In 3,4-PDCA, the dihedral angles between distinct π-conjugated planes span a wide range of 60–140°. This large distortion is attributed to the steric hindrance and intramolecular hydrogen bonding imposed by the ortho-disposed carboxylic acid groups on the pyridine ring. In contrast, the meta-arrangement of the two carboxylic acids in 3,5-PDCA reduces these interactions, giving rise to much smaller dihedral angles between the π-conjugated units. The closer planarity enhances the polarizability anisotropy of 3,5-PDCA relative to 3,4-PDCA, which is the primary reason for its superior birefringent response.


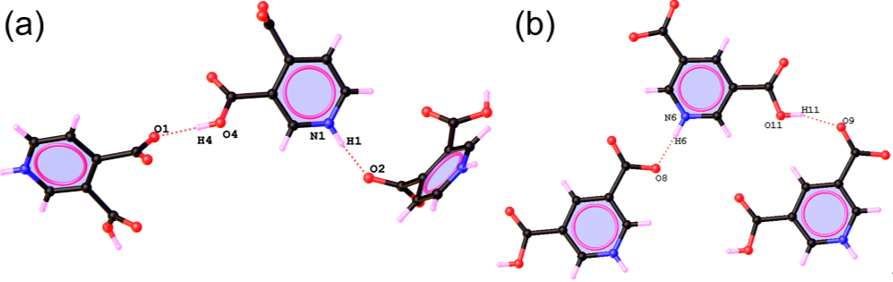


**Figure S4**. Hydrogen-bonding motifs in (a) **HOF-1** and (b) **HOF-2**. (a) **HOF-1**: intralayer N–H···O hydrogen bonds (N1–H1···O2, d_D–A_ = 2.659(5) Å) and interlayer O–H···O hydrogen bonds (O4–H4···O1, d_D–A_ = 2.510(5) Å). (b) **HOF-2**: intralayer N1–H1···O2 (d_D–A_ = 2.599(2) Å) and interlayer O4–H4···O1 (d_D–A_ = 2.528(3) Å). All distances are given as donor–acceptor separations.


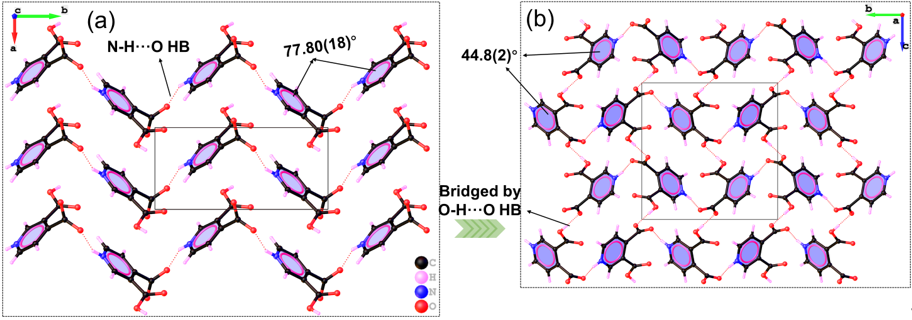


**Figure S5.** (a) One-dimensional [3,4-PDCA] hydrogen-bonded chain and (b) three-dimensional framework of **HOF-1**. Each 3,4-PDCA molecule is first linked by N–H···O hydrogen bonds, propagating along the b axis to generate a 1D chain. These chains run parallel within the ab plane, thereby forming a pseudo-2D layer. Subsequent O–H···O hydrogen bonds interconnect these layers into a 3D hydrogen-bonded network. Notably, the dihedral angle between adjacent pyridyl planes connected by N–H···O hydrogen bonds is 77.80(18)°, whereas that bridged by O–H···O hydrogen bonds is 44.8(2)°. The substantial torsion between π-conjugated units thus significantly diminishes the optical anisotropy of the crystal.


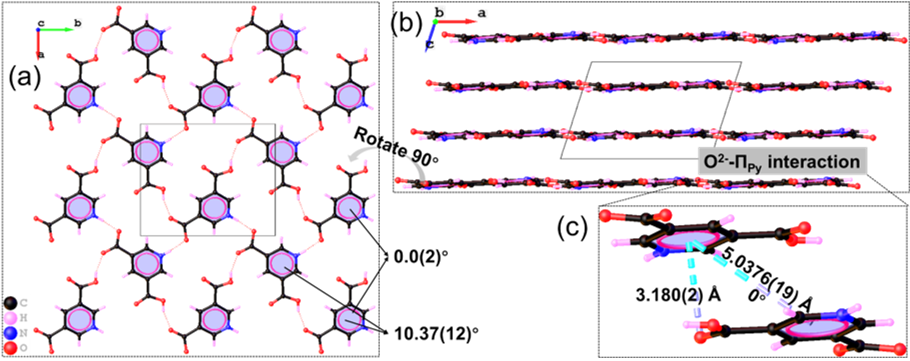


**Figure S6**. (a) Two-dimensional [3,5-PDCA] hydrogen-bonded layer, (b) overall structure of **HOF-2** viewed along the *c* axis, and (c) interlayer anion–π interactions. Adjacent 3,5-PDCA molecules are linked by N–H···O and O–H···O hydrogen bonds, generating 2D layers that lie parallel to the ab plane. These layers stack co-translationally along c, stabilized by anion–π contacts between the O2⁻ anion of a carboxylate and the pyridyl ring of the neighboring layer (anion–centroid distance = 3.180(2) Å). No π–π stacking is observed; the corresponding centroid–centroid separation is 5.0379(19) Å. Within each layer, the dihedral angle between hydrogen-bonded PDCA molecules is only 10.37(12)°, and π-conjugated units in adjacent layers are perfectly parallel. The pronounced contrast between strong intra-layer hydrogen bonding and weak, directional interlayer anion–π interactions gives rise to a highly anisotropic framework that favors large macroscopic optical birefringence.


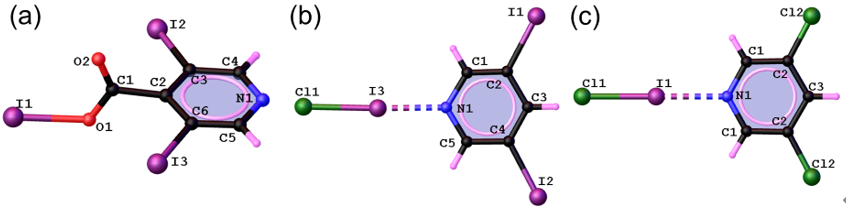


**Figure S7.** The asymmetric units of (a) 3,5-I₂Py-COOI, (b) 3,5-I₂Py-ICl, and (c) 3,5-Cl₂Py-ICl in **XOF-1**, **Hybrid-XOF-1** and **XB-HOF-1**, respectively. Key structural features are noted below:

In 3,5-I₂Py-COOI, all atoms occupy general positions. The dihedral angle between the pyridine ring and the COOI plane is 87.8(6)°.

In 3,5-I₂Py-ICl, all atoms also occupy general positions.

In 3,5-Cl₂Py-ICl, atoms C1, C2, and Cl2 are in general positions, whereas atoms Cl1, I2, N1, and C3 are located on a crystallographic mirror plane (m).


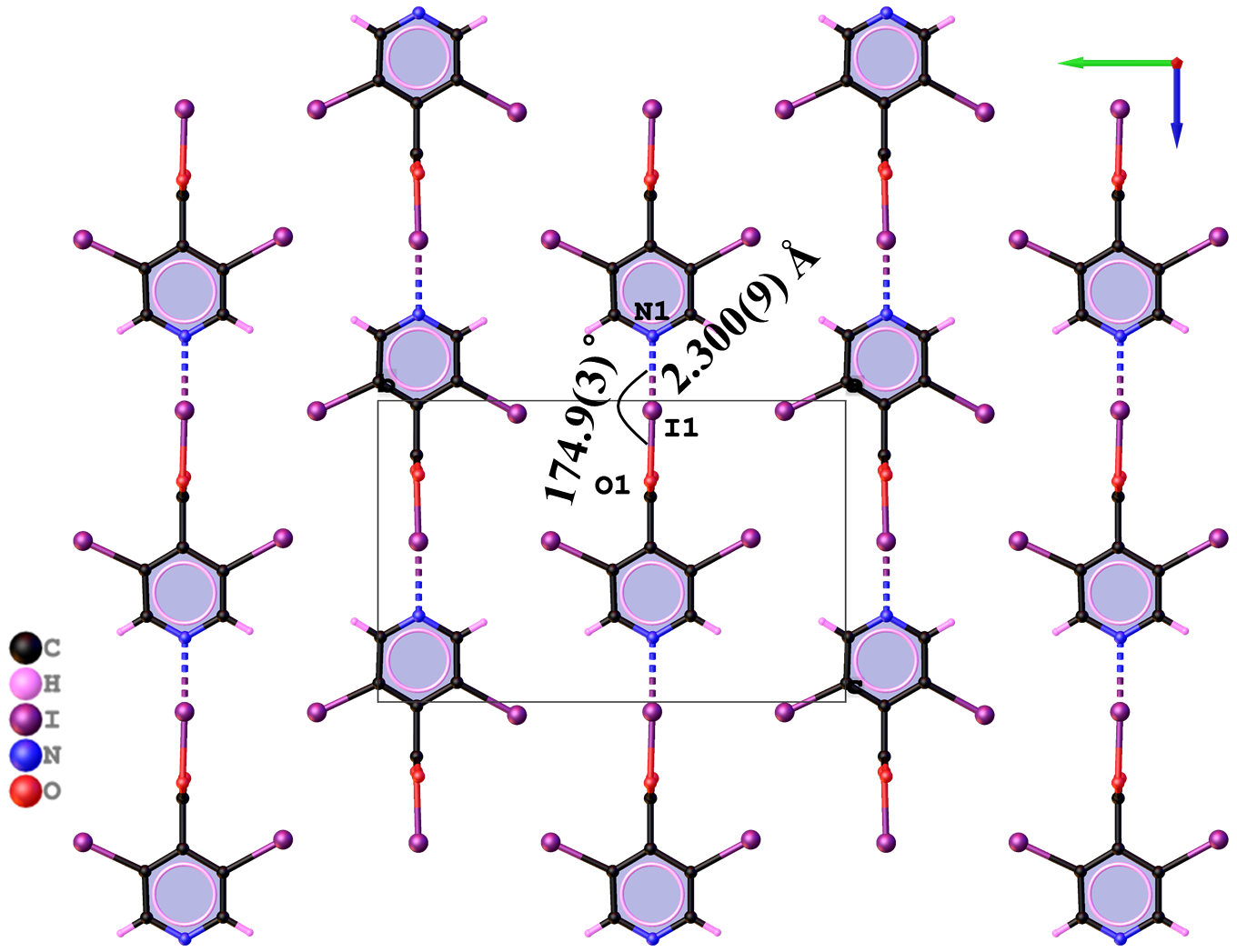


**Figure S8.** Packing diagram of 3,5-I₂Py-COOI viewed along the crystallographic *a*-axis, illustrating the formation of a 2D pseudo-layered structure. Within this architecture, adjacent 3,5-I₂Py-COOI units are linked by N···I–O halogen bonds to form 1D chains running parallel to the c-axis. These chains are further assembled into layers through weak dispersion interactions.


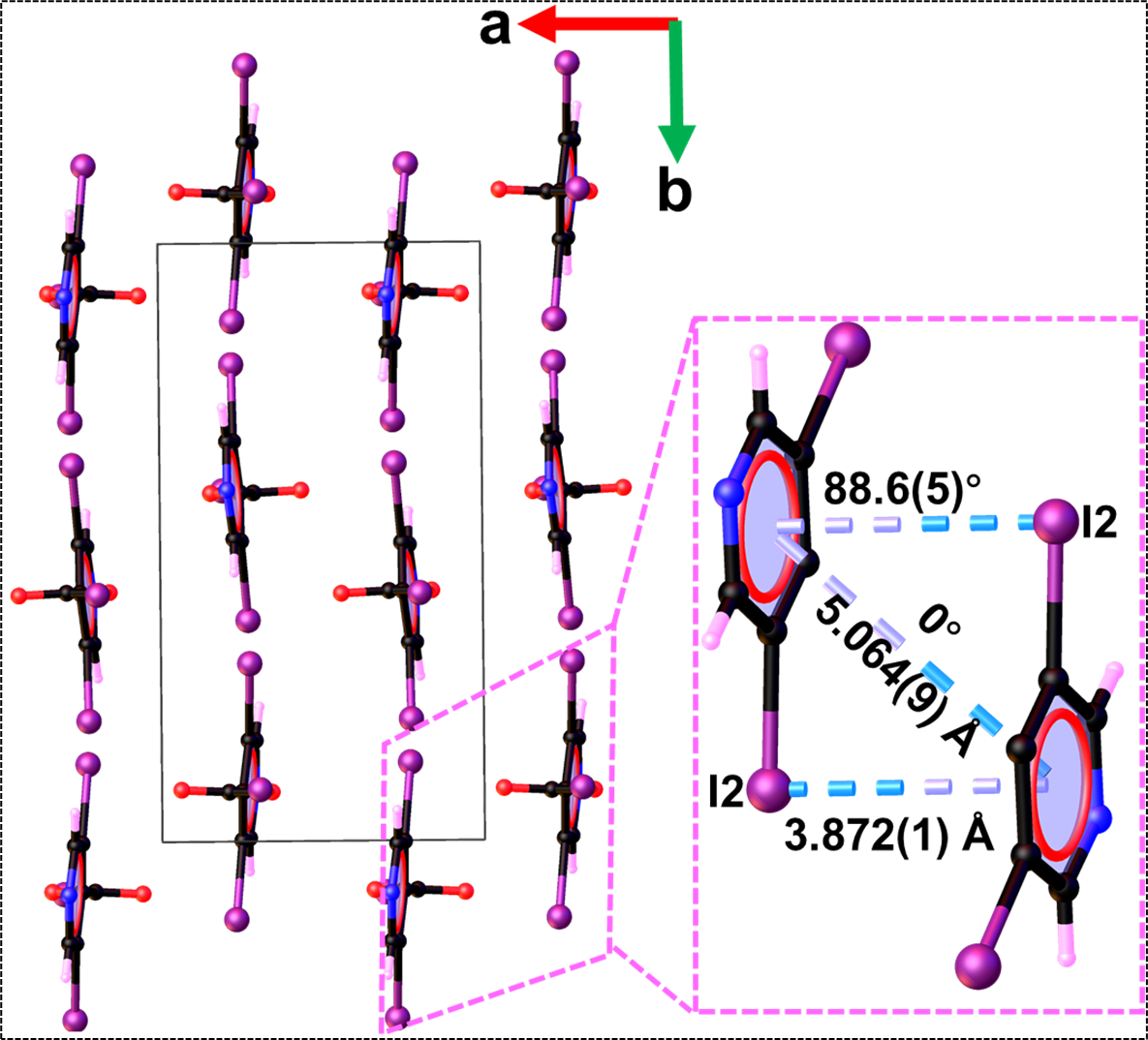


**Figure S9.** Packing diagram illustrating the 3D supramolecular architecture of **XOF-1**, viewed along the crystallographic *c*-axis.


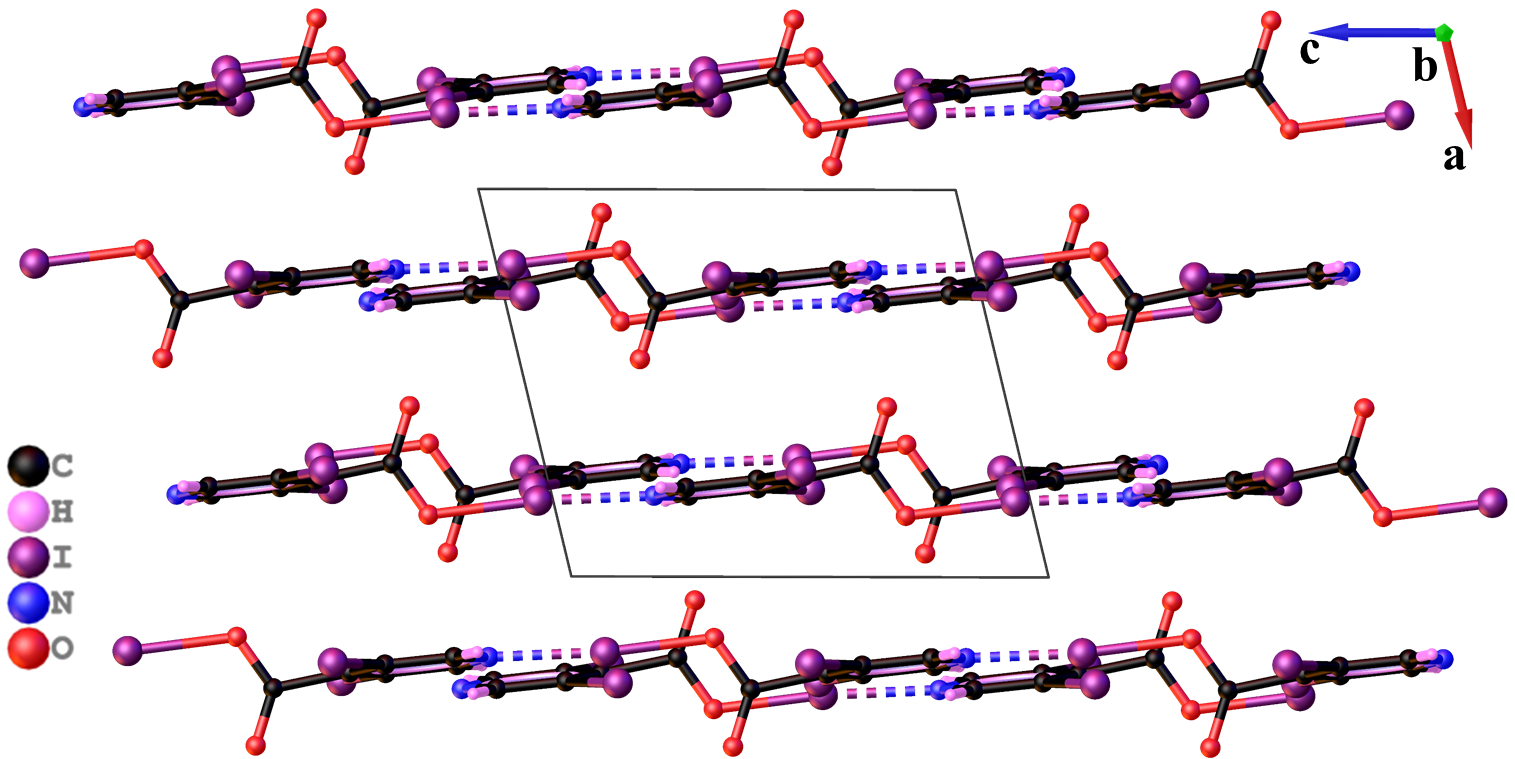


**Figure S10**. Packing diagram illustrating the 3D supramolecular architecture of **XOF-1**, viewed along the crystallographic *b*-axis. The 2D pseudo-layers (shown in Figure S7) are stacked in a parallel fashion. The interlayer packing is primarily governed by anion–π interactions between the iodide centers and the pyridine rings, characterized by an I⁻···Py(centroid) distance of 3.872(1) Å. In contrast, the potential π–π stacking interactions between opposing pyridine rings of adjacent layers are negligible, as indicated by a large centroid-to-centroid distance of 5.064(9) Å, despite a parallel orientation (dihedral angle of 0°).


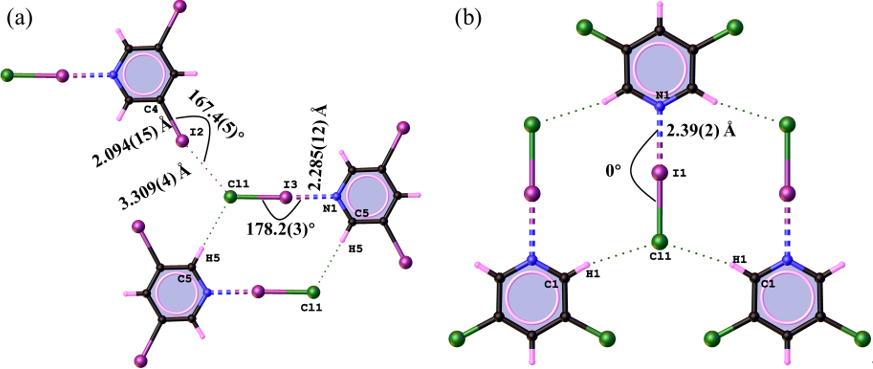


**Figure S11**. Halogen- and hydrogen-bonding interactions in (a) **Hybrid-XOF-1** and (b) **XB-HOF-1**.


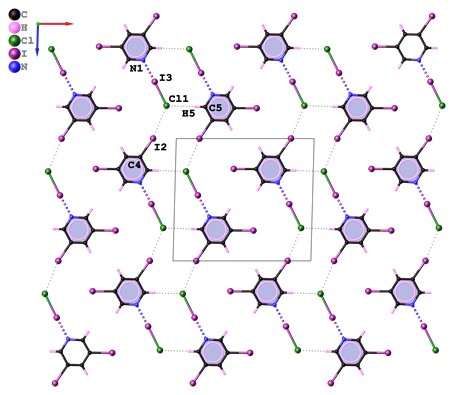


**Figure S12.** Packing diagram of 3,5-I_2_Py-ICl viewed along the crystallographic *c*-axis, illustrating its 2D pseudo-layered structure in **Hybrid-XOF-1**. The 1D chains are arranged parallel to each other and lie coplanar within the ac-plane. These chains are interconnected by weak C5–H5···Cl1 hydrogen bonds [D(D-A) = 3.593(17) Å] to form the layers. Crystallographic axes are indicated by colored arrows: *a* (red), *b* (blue), and *c* (green).


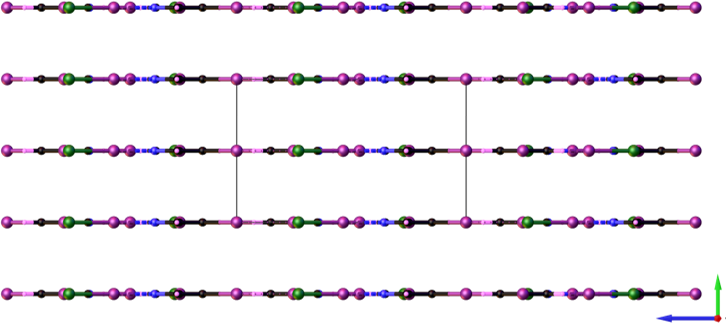


**Figure S13**. Perspective view of the 3D **Hybrid-XOF-1** framework along the crystallographic *a* axis. Crystallographic axes are indicated by colored arrows: *a* (red), *b* (blue), and *c* (green).


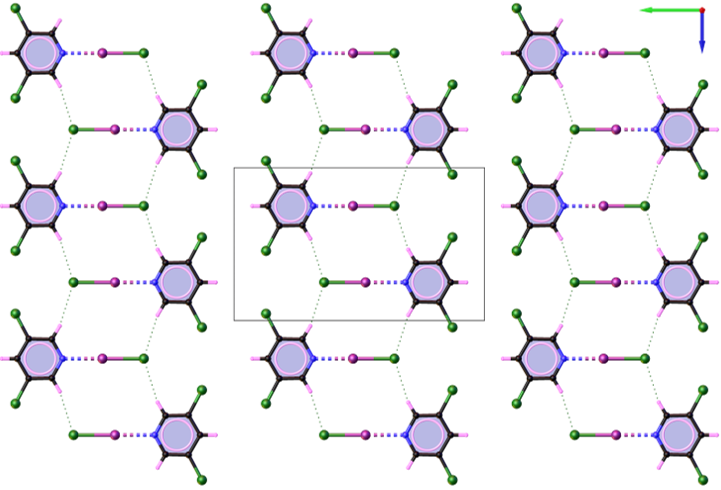


**Figure S14.** Packing diagram of 3,5-Cl₂Py-ICl viewed along the crystallographic *a*-axis in **XB-HOF-1**, illustrating its 2D pseudo-layered structure. The constituent 1D chains are arranged parallel to one another and lie coplanar within the *bc*-plane. Crystallographic axes are indicated by colored arrows: *a* (red), *b* (blue), and *c* (green).


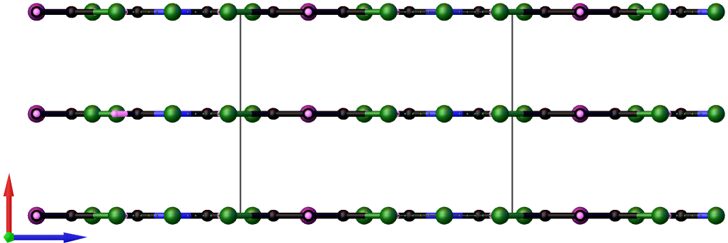


**Figure S15**. Perspective view of the 3D **XB-HOF-1** framework along the crystallographic *b* axis. Crystallographic axes are indicated by colored arrows: *a* (red), *b* (blue), and *c* (green).


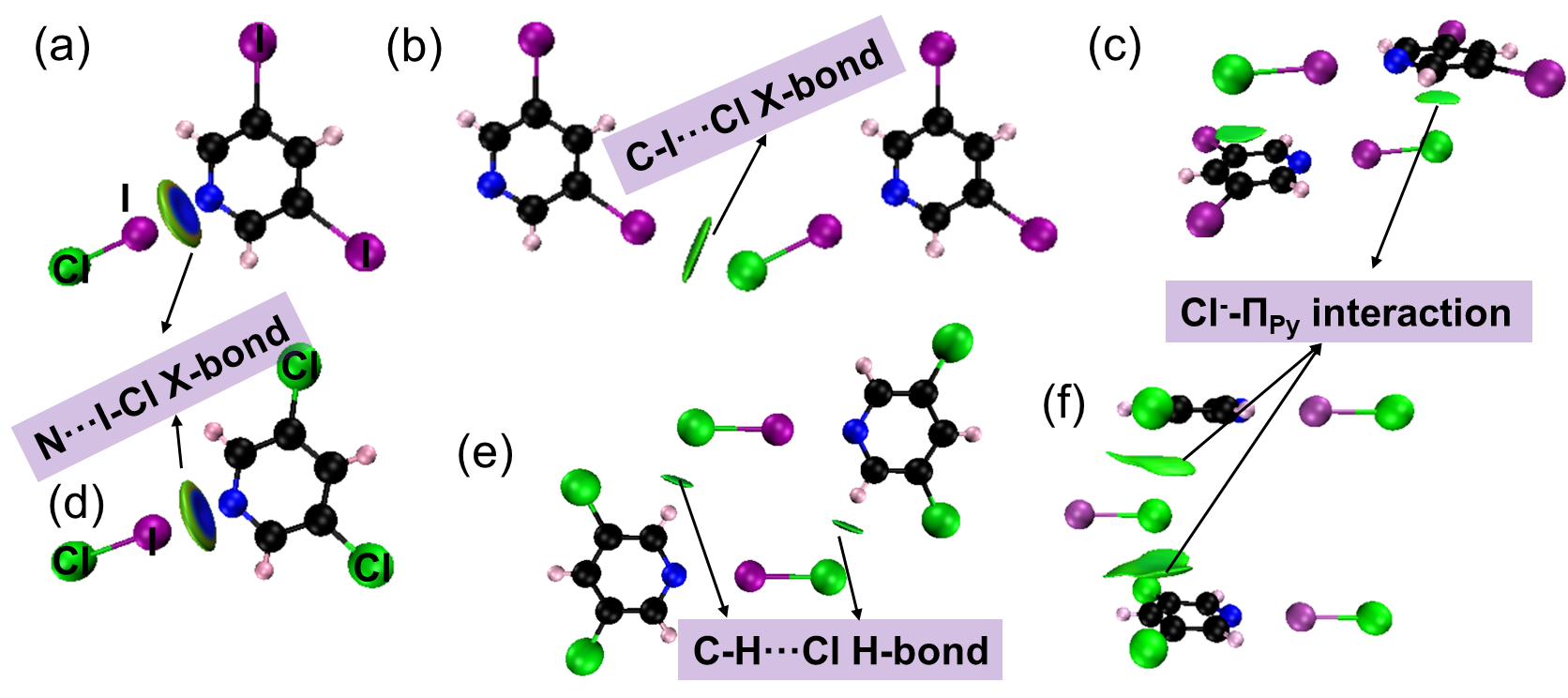


**Figure S16**. IGMH isosurface plots for 3,5-I_2_Py-ICl (a, c, e) and 3,5-Cl_2_Py-ICl (b, d, f) in **Hybrid-XOF-1** and **XB-HOF-1**, respectively. Computational analysis using the Independent Gradient Model based on Hirshfeld partitioning (IGMH) provides visual and quantitative support for our structural observations. The IGMH isosurface plots clearly delineate the hierarchy of interactions governing the crystal assembly. For both 3,5-I₂Py-ICl and 3,5-Cl₂Py-ICl, a large, blue-green-centered isosurface appears between the pyridine nitrogen and the iodine of the ICl moiety, which is characteristic of a strong, three-center four-electron N···I–Cl halogen bond. In stark contrast, a distinct green isosurface is observed between the chloride atom of the ICl unit and the center of the pyridine ring in the adjacent layer, confirming that the interlayer packing is dominated by anion-π van der Waals forces rather than π-π stacking. Furthermore, the analysis highlights a critical difference between the two compounds: in 3,5-I₂Py-ICl, an additional green isosurface exists between the ring-iodine atom and the chlorine of a neighboring ICl unit, visually confirming the presence of a weak C–I···Cl halogen bond. In 3,5-Cl_2_PY-ICl, the presence of a green isosurface between the H atom on the pyridine ring and the Cl atom in the neighboring ICl unit visually verifies the presence of weak C-H···Cl hydrogen bonds.


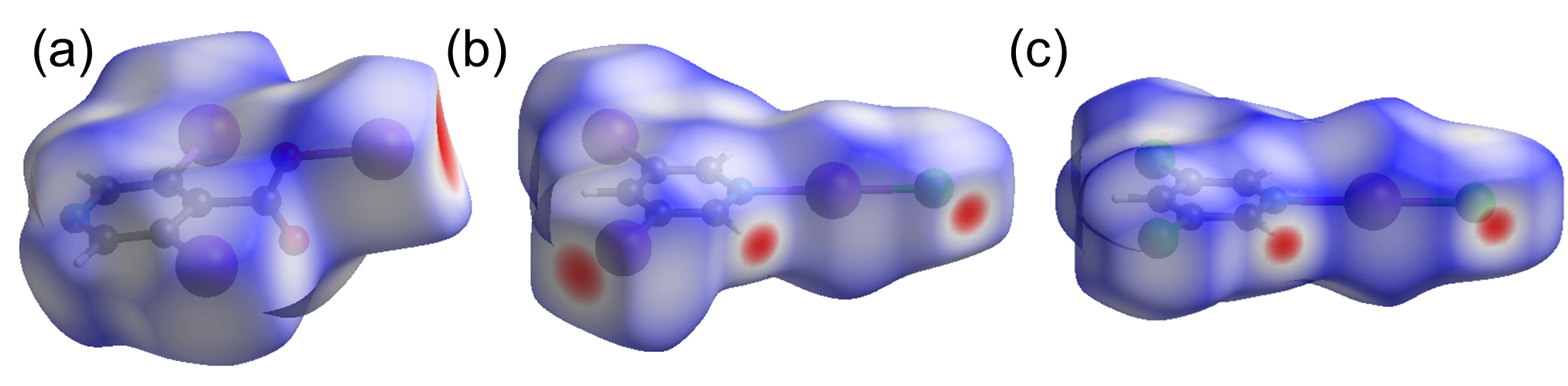


**Figure S17.** Hirshfeld surfaces mapped with d_norm_ for (a) 3,5-I₂Py-COOI, (b) 3,5-I₂Py-ICl, and (c) 3,5-Cl₂Py-ICl. The d_norm_ maps visualize intermolecular contacts, where red regions indicate contacts shorter than the sum of van der Waals radii, white regions represent contacts of similar length, and blue regions correspond to longer contacts.

(i) For 3,5-I₂Py-COOI, the prominent red regions on the iodine atoms are indicative of strong N–I···O halogen bonds that connect adjacent molecules.

(ii) In 3,5-I₂Py-ICl, the surface reveals the presence of both weak C–I···Cl halogen bonds and non-negligible C–H···Cl hydrogen bonds as key intermolecular interactions.

(iii) In contrast, the surface for 3,5-Cl₂Py-ICl shows that potential C–Cl···Cl halogen bonds are negligible, with the packing being predominantly stabilized by C–H···Cl weak hydrogen bonds.


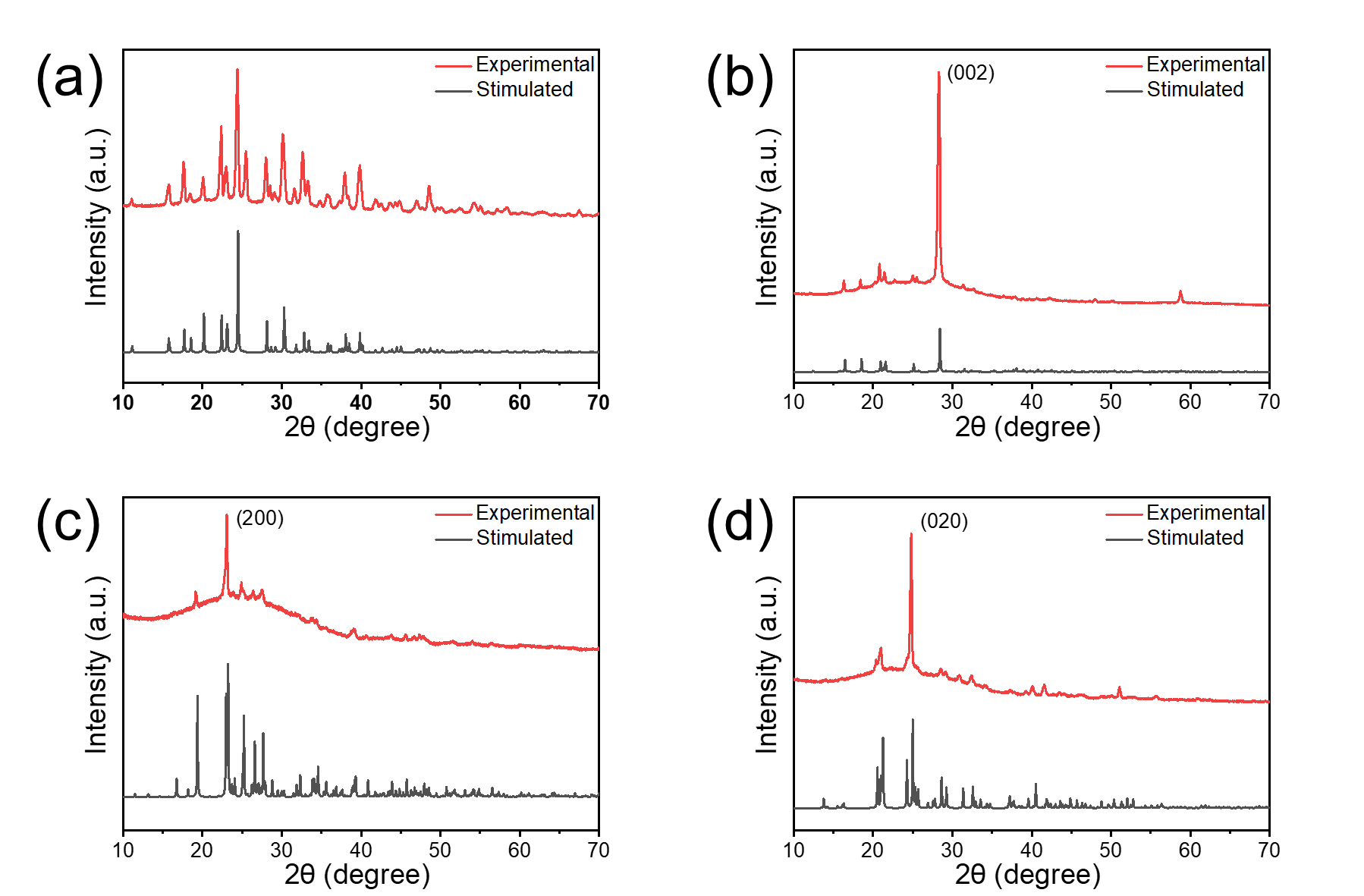


**Figure S18.** Experimental and simulated PXRD patterns for (a) **HOF-1**, (b) **HOF-2**, (c) **XOF-1**, and (d) **Hybrid-XOF-1**. When these traces are combined with the PXRD data for **XB-HOF-1** shown in Figure 3a, two conclusions emerge. First, the close agreement between experimental and simulated peaks confirms the phase purity of all samples. Second, **HOF-2**, **XOF-1**, **Hybrid-XOF-1**, and **XB-HOF-1** exhibit pronounced preferred orientation; the most intense reflection in each pattern corresponds to the (002), (200), (020) and (200) planes that contain the 2D [3,5-PDCA] hydrogen-bonded layers or pseudo-layers in **XOF-1**, **Hybrid-XOF-1** and **XB-HOF-1**. This observation indicates that the anion–π interactions responsible for interlayer cohesion are relatively weak and are preferentially disrupted during sample grinding.


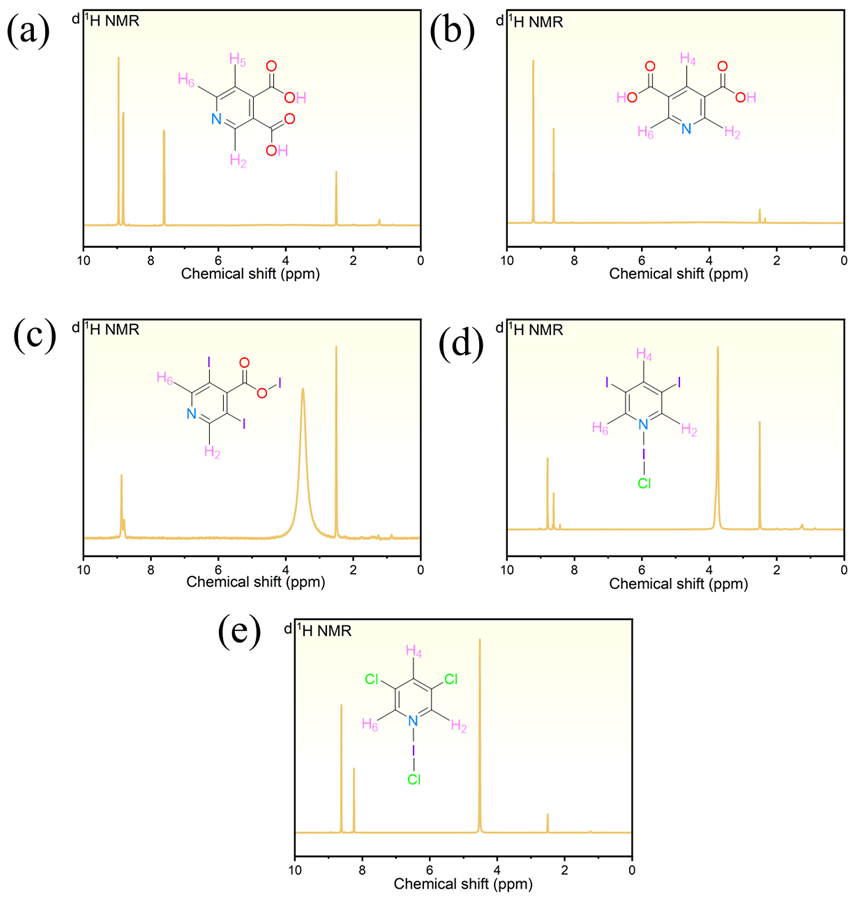


**Figure S19**. ¹H NMR spectra (400 MHz, DMSO-d_6_, 298 K) of (a) 3,4-PDCA, (b) 3,5-PDCA, (c) 3,5-I_2_Py-COOI, (d) 3,5-I_2_Py-ICl, and (e) 3,5-Cl_2_Py-ICl. The residual solvent peak appears at δ 2.50 ppm. (a) 3,4-PDCA: The three pyridyl protons are chemically non-equivalent. The spectrum shows three sets of signals: a singlet at δ 8.95 ppm (H_2_, s), a doublet at δ 8.83 ppm (H_6_, d, J = 4.0 Hz), and a doublet at δ 7.62 ppm (H_5_, d, J = 8.0 Hz). (b) 3,5-PDCA: Owing to C_2v_ molecular symmetry, the pyridyl protons are chemically equivalent in two sets, resonating as singlets at δ 9.22 ppm (H_4_, s) and δ 8.22 ppm (H_2_/H_6_, s). (c) 3,5-I_2_Py-COOI: The two pyridyl protons (H_2_ and H_6_) are chemically non-equivalent due to the asymmetric -COOI substituent, giving two doublets at δ 8.87 ppm (d, J = 2.1 Hz) and δ 8.79 ppm (d, J = 2.1 Hz). (d) 3,5-I_2_Py-ICl: Owing to C_2v_ molecular symmetry, the two pyridyl protons are chemically equivalent and resonate as a singlet at δ 8.79 ppm (H_4_, s). The signal at δ 8.26 ppm is assigned to H_2_/H_6_ (s). (e) 3,5-Cl_2_Py-ICl: Owing to C_2v_ molecular symmetry, the two pyridyl protons are chemically equivalent and resonate as a singlet at δ 8.62 ppm (H_4_, s). The signal at δ 8.25 ppm is assigned to H_2_/H_6_ (s).


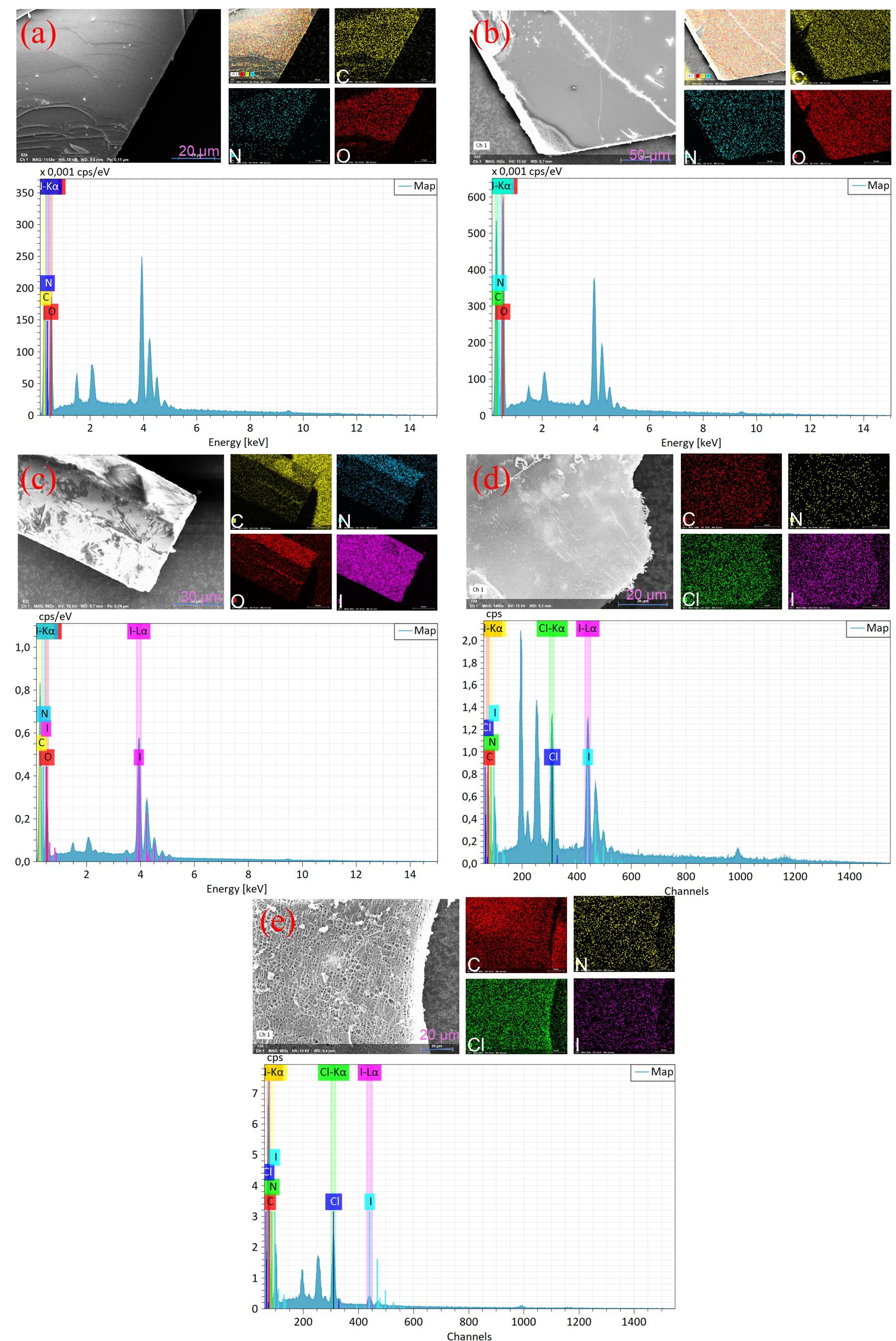


**Figure S20.** Field-emission scanning electron microscope (FESEM) analyses **HOF-1** (a), **HOF-2** (b), **XOF-1** (c), **Hybrid-XOF-1** (d) and **XB-HOF-1** (e).


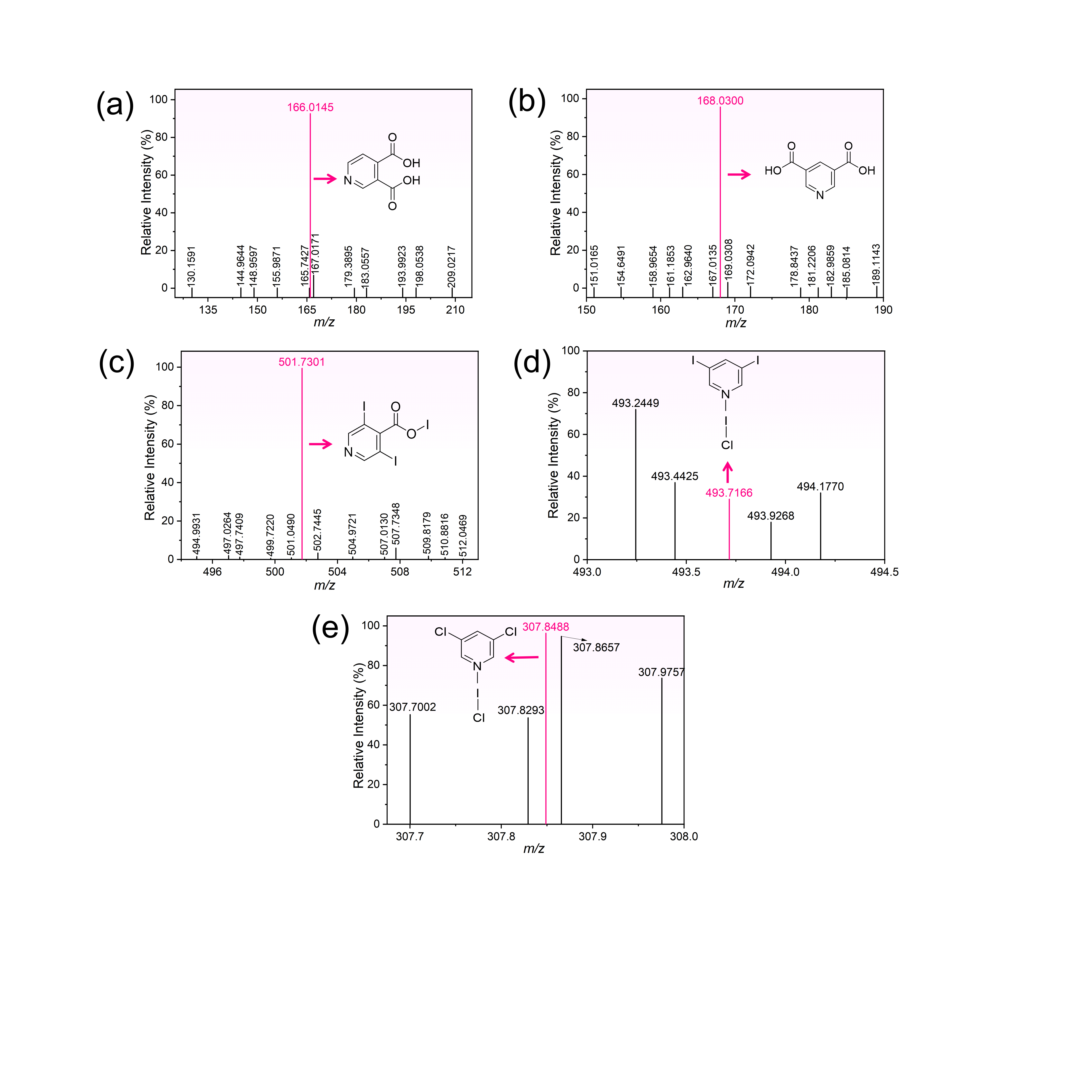


**Figure S21**. Based on high-resolution mass spectrometry analysis, the molecular formulas of the respective compounds were accurately assigned as follows:

(a) In negative ion electrospray ionization mode, the quasi-molecular ion peak at m/z = 166.0145, with an elemental composition of C_7_H_2_NO_4_ (mass error +0.5 mDa), corresponds to the deprotonated ion [M−H]^-^ of 3,4-PDCA, confirming its molecular formula as C_7_H_3_NO_4_. (b) In positive ion mode, the peak at  m/z = 168.0300, matching the composition C_7_H_4_NO_4_ (mass error +0.3 mDa), is attributed to the protonated ion [M+H]^+^ of 3,4-PDCA, verifying its molecular formula as C_7_H_3_NO_4_. (c) In positive ion mode, the ion signal at  m/z = 501.7301, with an elemental composition of C_6_H_3_NO_2_I_3_ (mass error +0.3 mDa), is consistent with the protonated molecule [M+H]^+^ of 3,5-I_2_Py-COOI, confirming the molecular formula as C_6_H_2_NO_2_I_3_. (d) Extracted ion chromatogram analysis in positive ion mode revealed a mass signal at m/z = 493.7166, corresponding to the elemental composition C_5_H_4_NClI_3_ (mass error 0.0 mDa), which is attributed to the [M+H]^+^ ion of a structural isomer of 3,5-I_2_Py-ICl, with the molecular formula determined as C_5_H_3_NClI_3_.(e) In negative ion mode, the quasi-molecular ion peak at m/z = 307.8293, matching the composition C_5_H_2_NCl_3_I (mass error −0.5 mDa), corresponds to the deprotonated ion [M−H]^-^ of 3,5-Cl_2_Py-ICl, confirming its molecular formula as C_5_H_3_NCl_3_I .


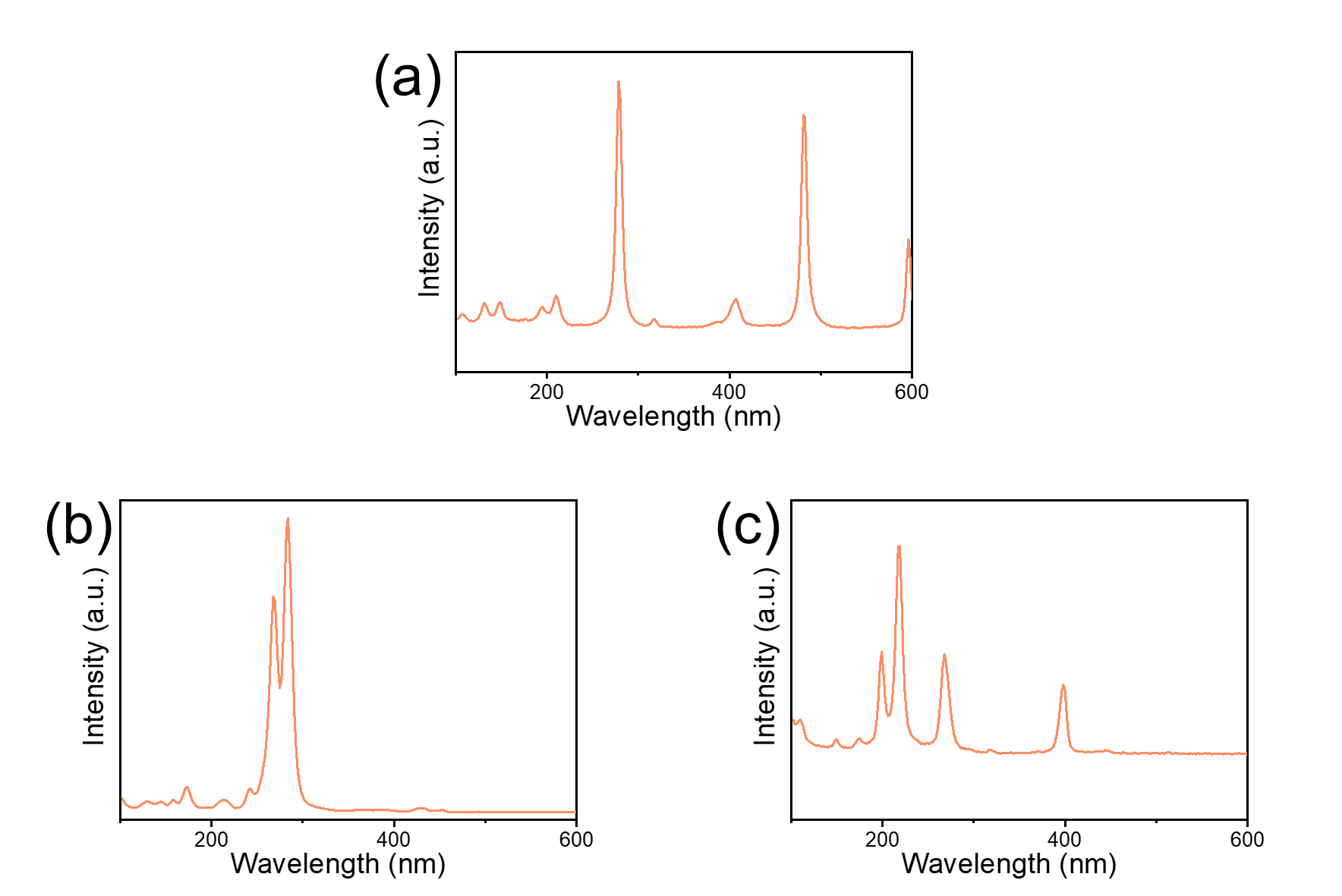


**Figure S22.** Raman spectra of (a) **XOF-1**, (b) **Hybrid-XOF-1**, and (c) **XB-HOF-1** collected at 298 K with 532 nm excitation.


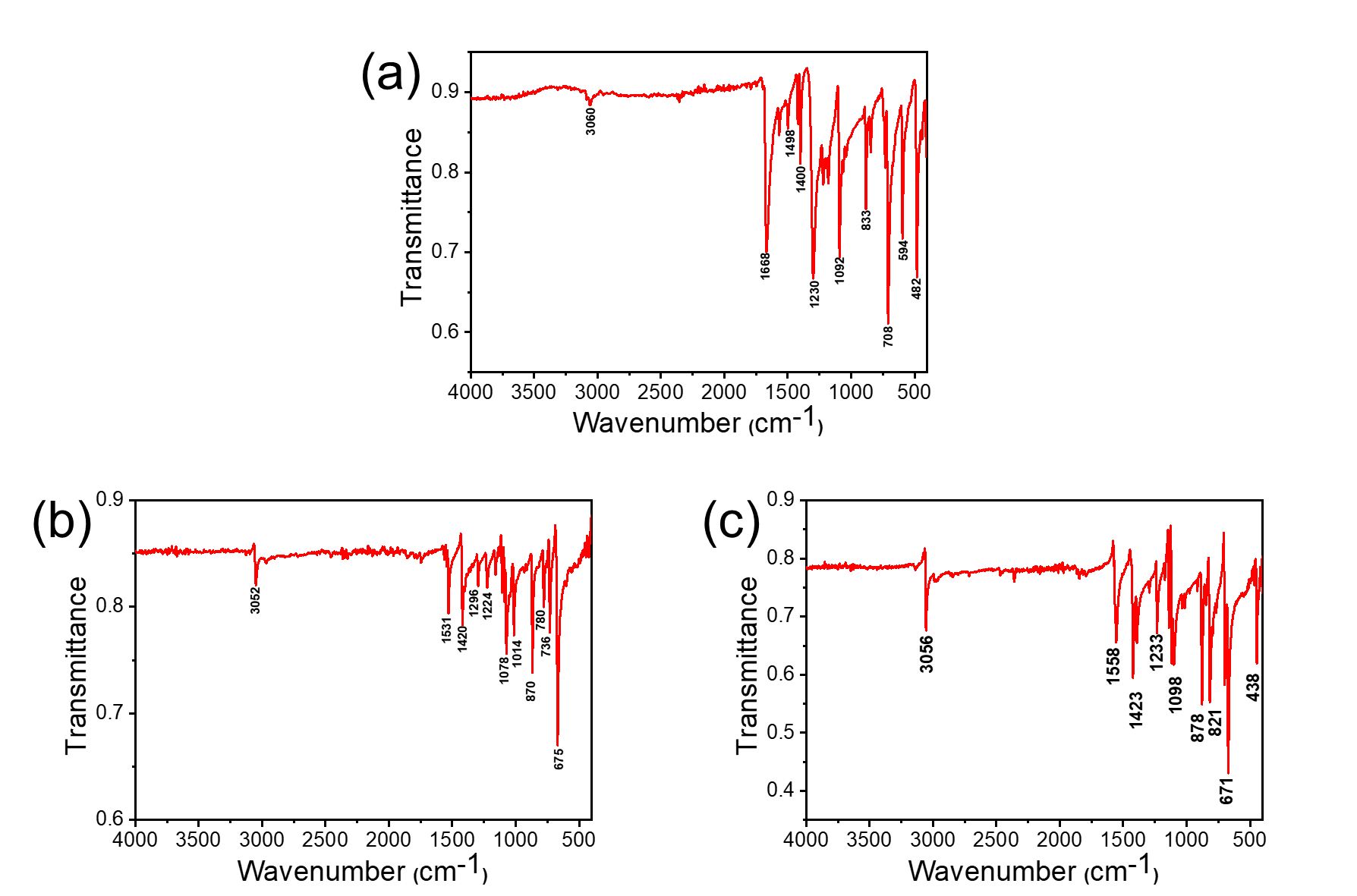


**Figure S23.** IR spectra for **XOF-1** (a), **Hybrid-XOF-1** (b) and **XB-HOF-1** (c).


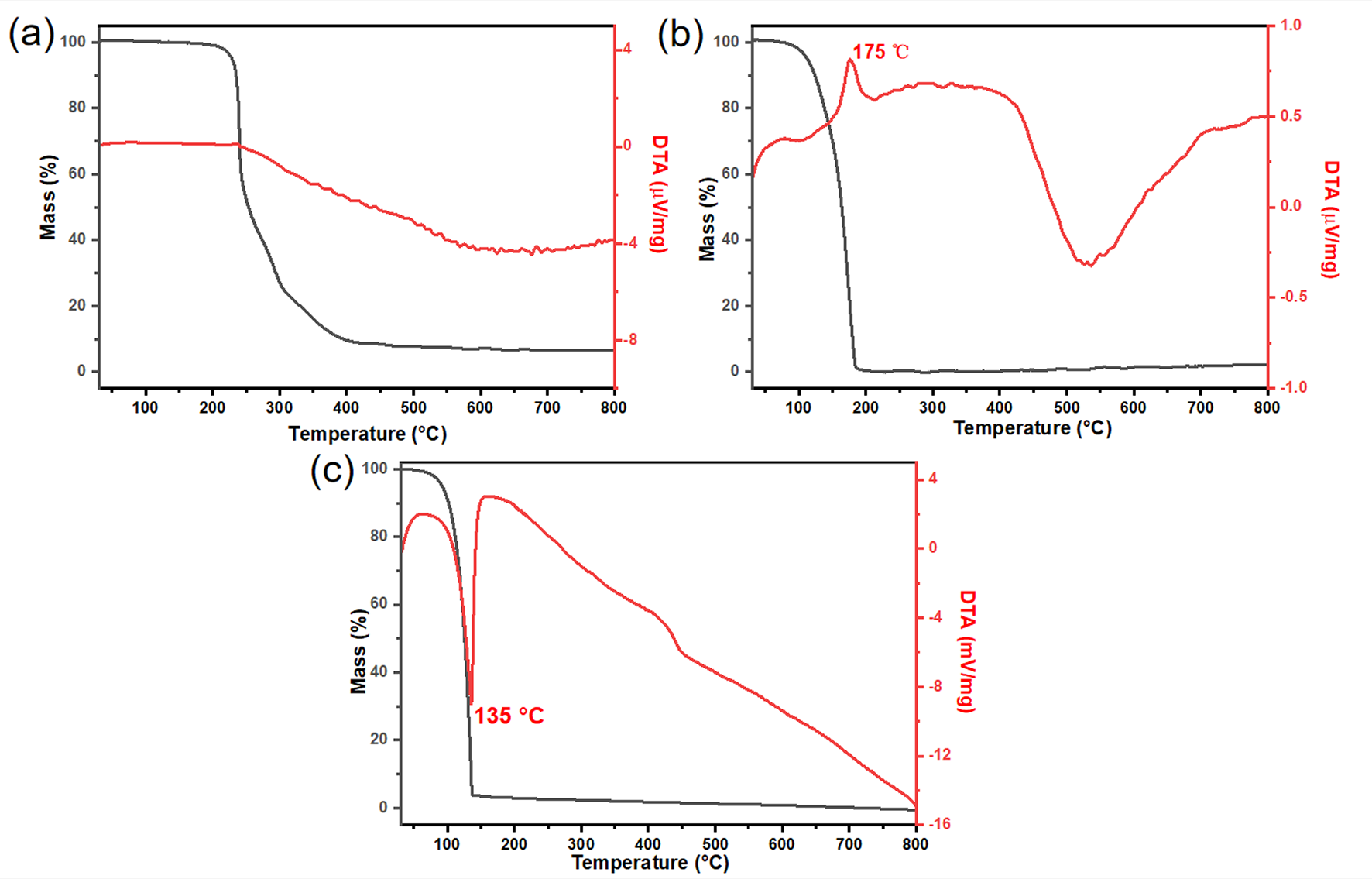


**Figure S24.**TG and DTA curves for **XOF-1** (a), **Hybrid-XOF-1** (b) and **XB-HOF-1** (c).


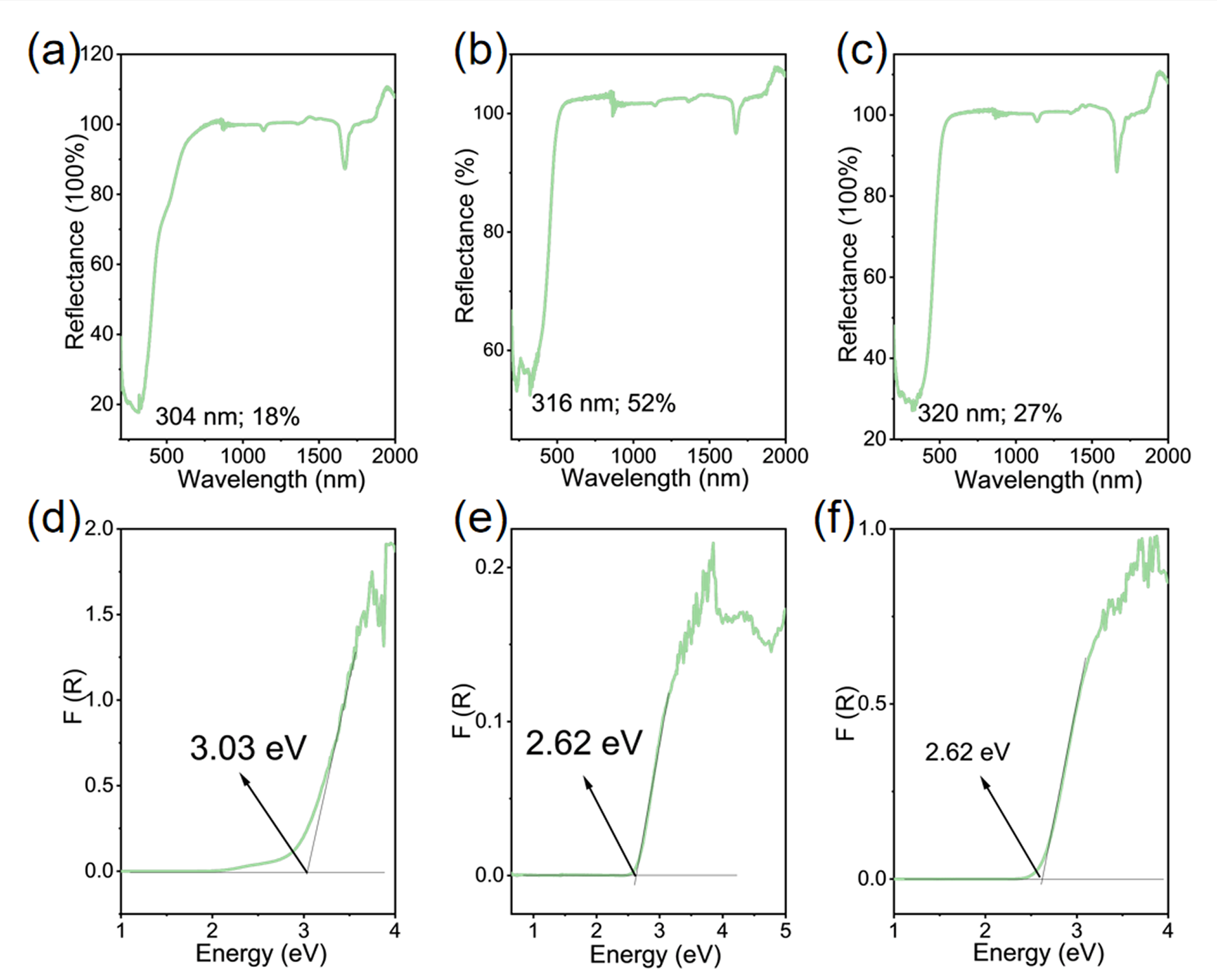


**Figure S25.** UV-Vis-IR spectra (a-c) and optical bandgaps (d-f) for **XOF-1**, **Hybrid-XOF-1** and **XB-HOF-1**.


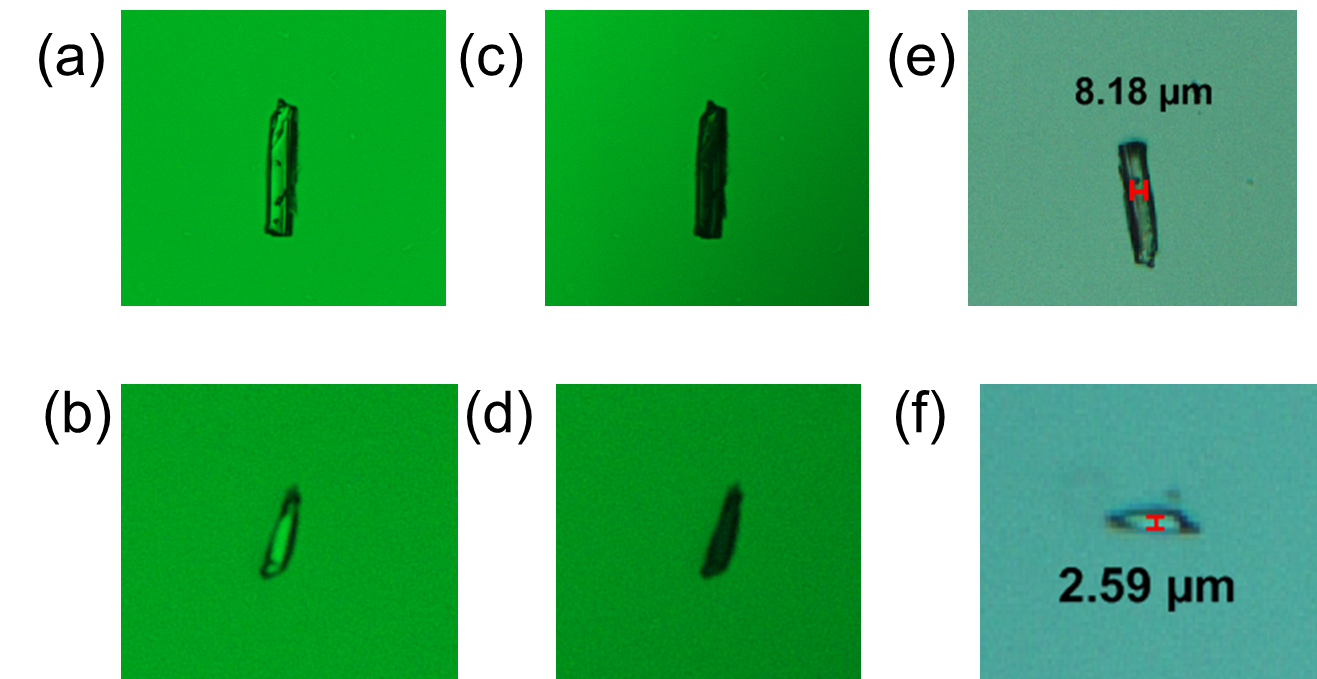


**Figure S26.** Birefringence characterization of the parent molecules, **HOF-1** and **HOF-2**. (a, c, e) correspond to a **HOF-1** crystal, and (b, d, f) correspond to a **HOF-2** crystal. (a, b) Crystals oriented at their maximum brightness positions. (c, d) The same crystals after adjustment of a Berek compensator to achieve optical compensation. (e, f) Measurement of the respective crystal thicknesses (*t*).


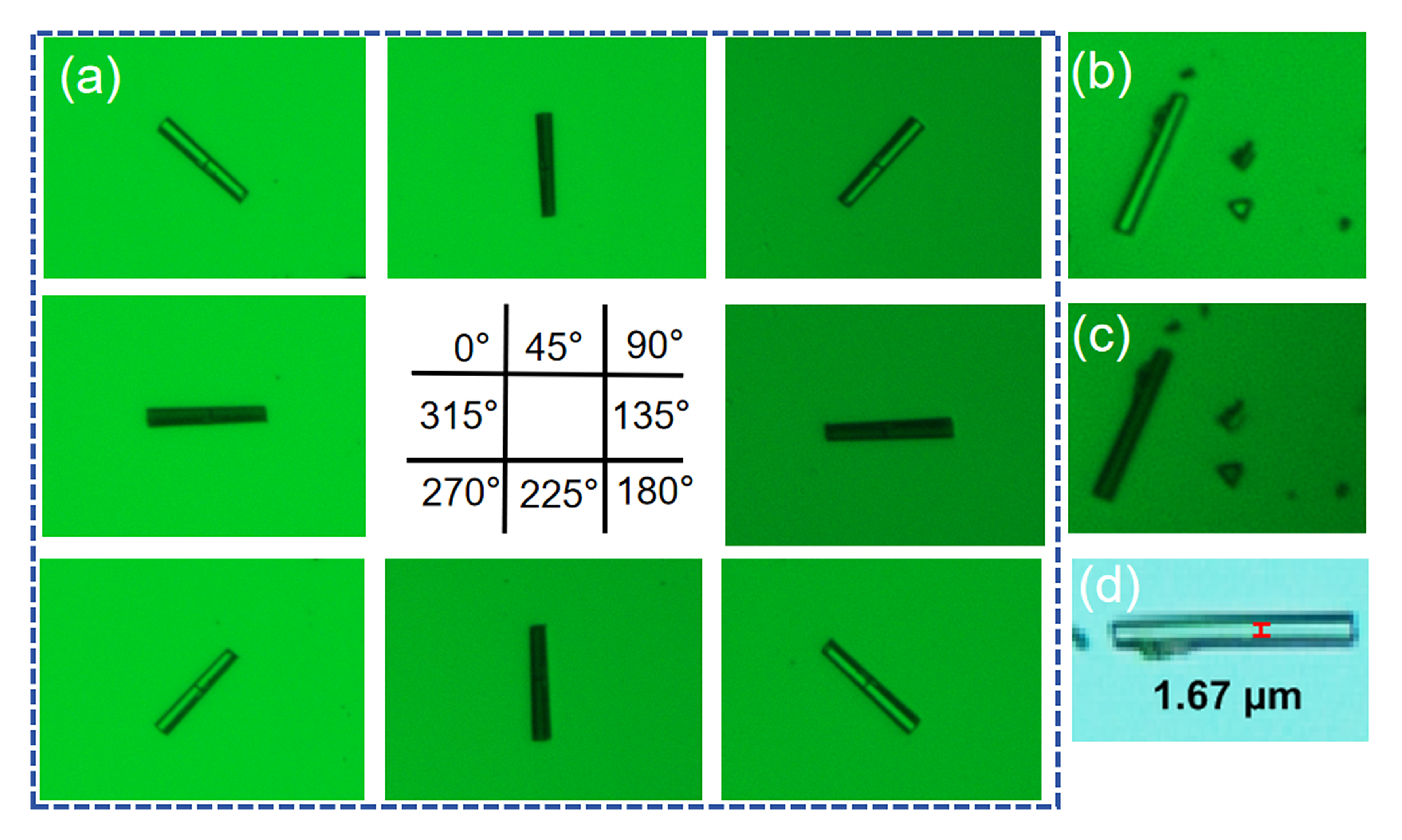


**Figure S27.** Optical characterization of a single crystal of **XOF-1** for birefringence measurement. (a) Polarized optical micrograph of the crystal under crossed polarizers, exhibiting distinct brightness variations and extinction upon rotation. (b) The crystal oriented at the 45° position to achieve maximum brightness. (c) The same crystal after insertion and adjustment of a Berek compensator, showing complete optical extinction (compensation). (d) Measurement of the crystal's thickness (*t*).


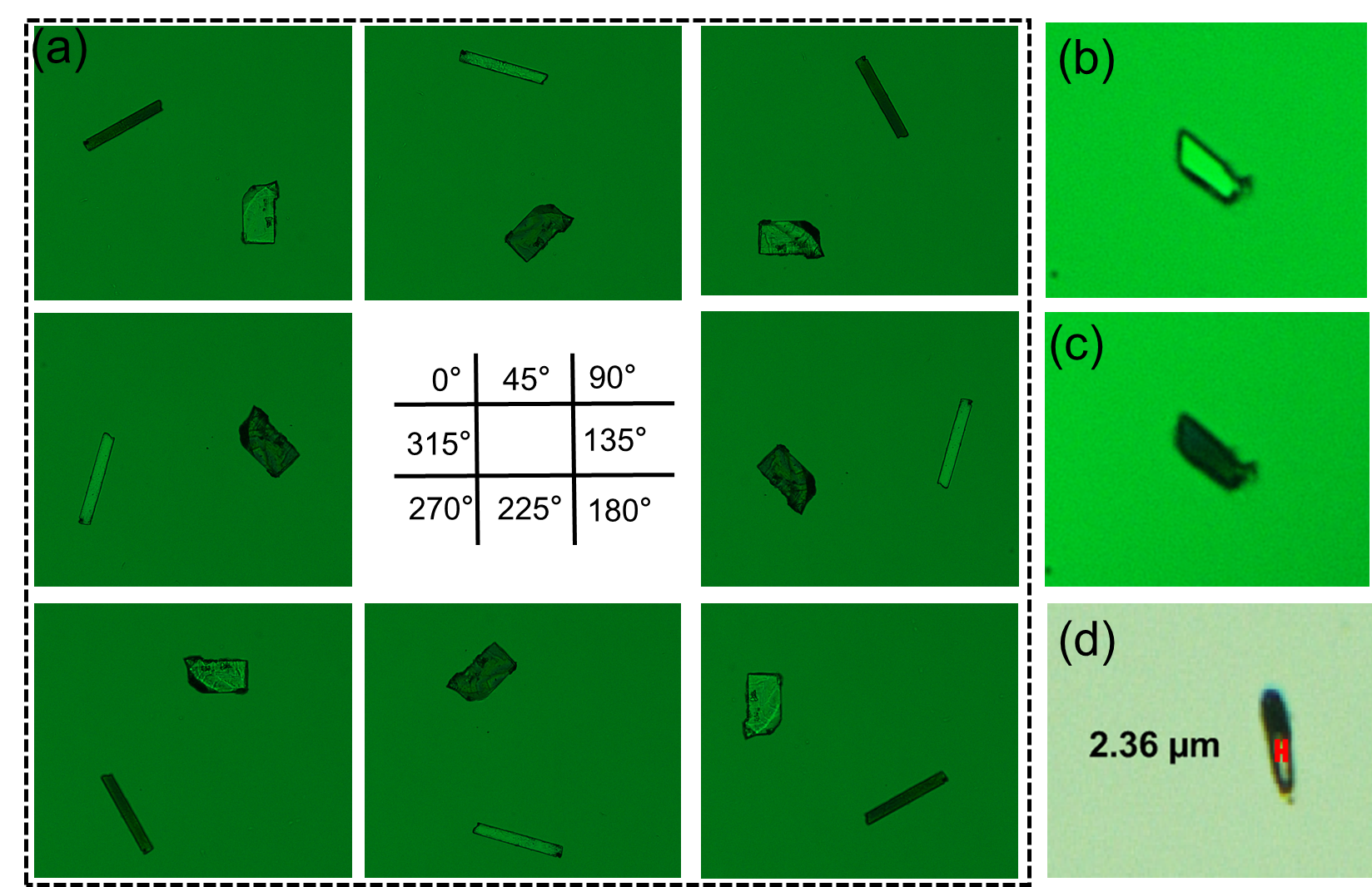


**Figure S28.** Optical characterization of a single crystal of **Hybrid-XOF-1** for birefringence measurement. (a) Polarized optical micrograph of the crystal under crossed polarizers, exhibiting distinct brightness variations and extinction upon rotation. (b) The crystal oriented at the 45° position to achieve maximum brightness. (c) The same crystal after insertion and adjustment of a Berek compensator, showing complete optical extinction (compensation). (d) Measurement of the crystal's thickness (t).


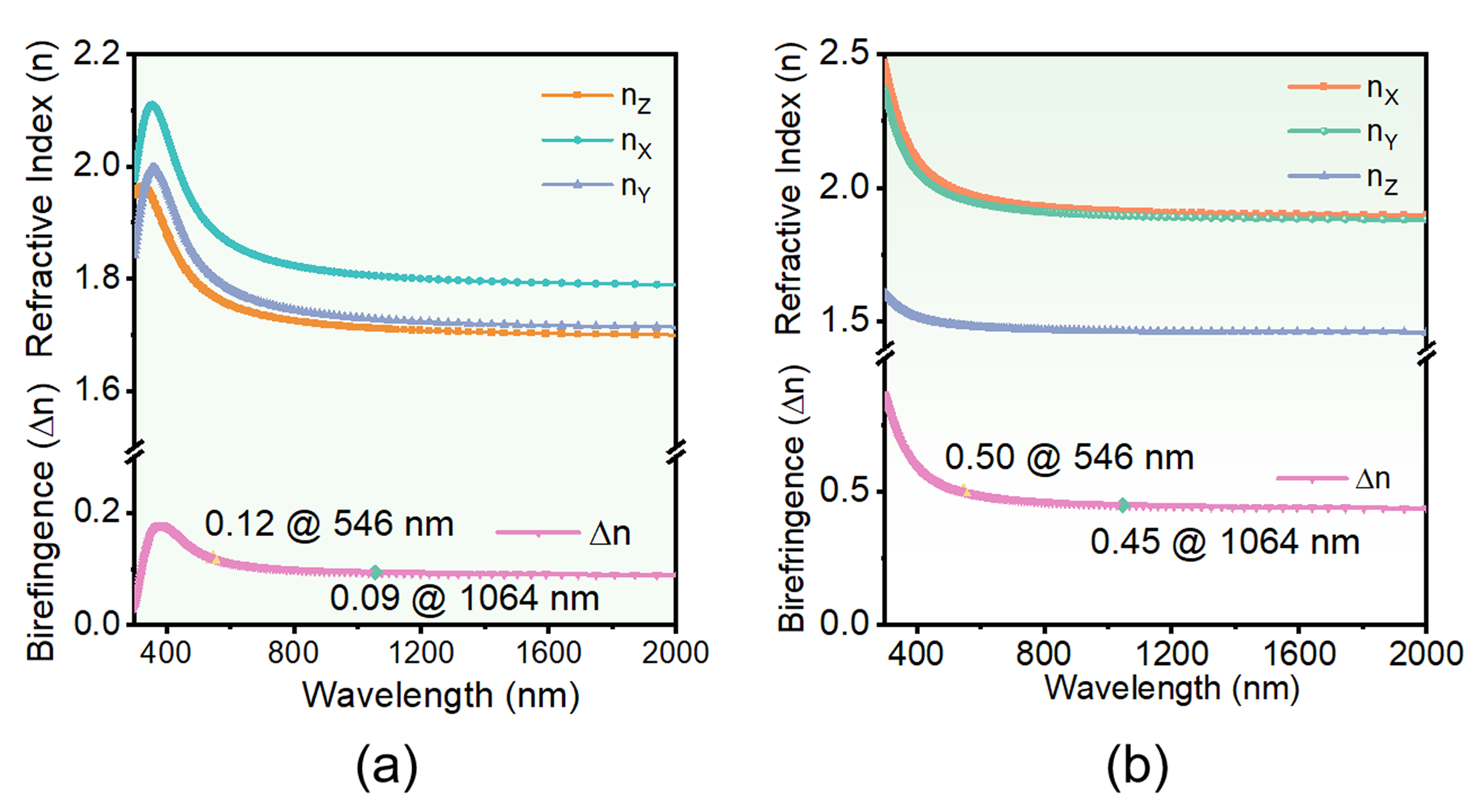


**Figure S29**. The calculated refractive index and birefringence of **HOF-1** (a) and **HOF-2** (b).


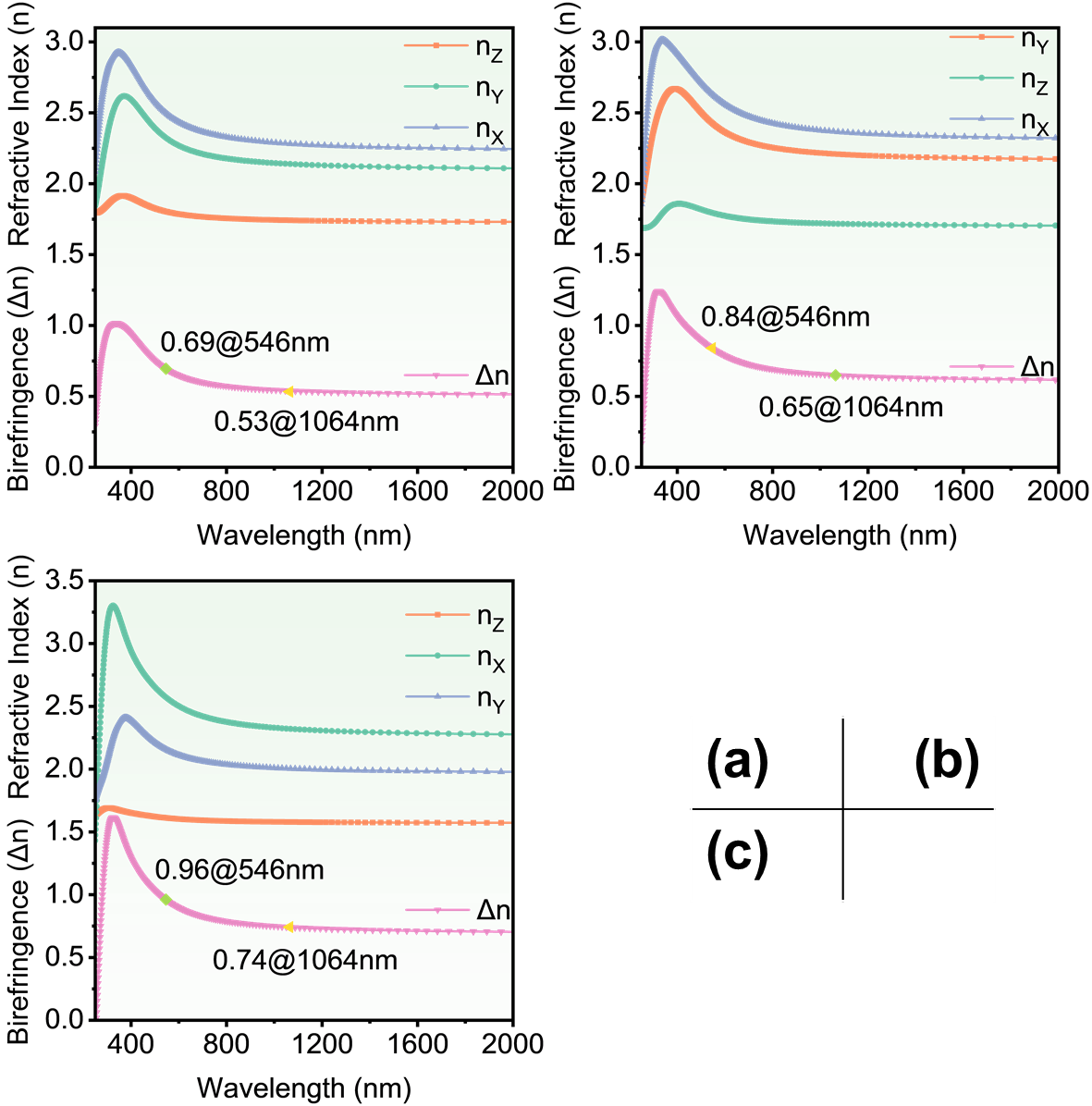


**Figure S30.** The calculated refractive index and birefringence of **XOF-1** (a), **Hybrid-XOF-1** (b) and **XB-HOF-1** (c).


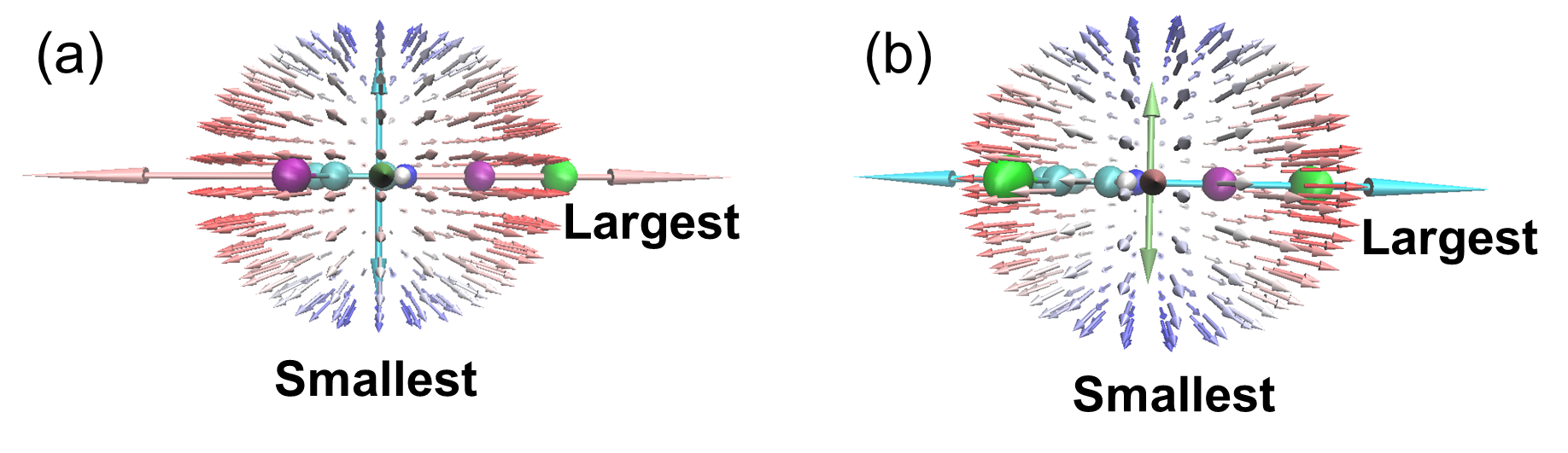


**Figure S31.** Unit sphere representation of polarizability for 3,5-I_2_Py-ICl (a) and 3,4-I_2_Py-ICl (b) in **Hybrid-XOF-1** and **XB-HOF-1**, respectively, under a static electric field.


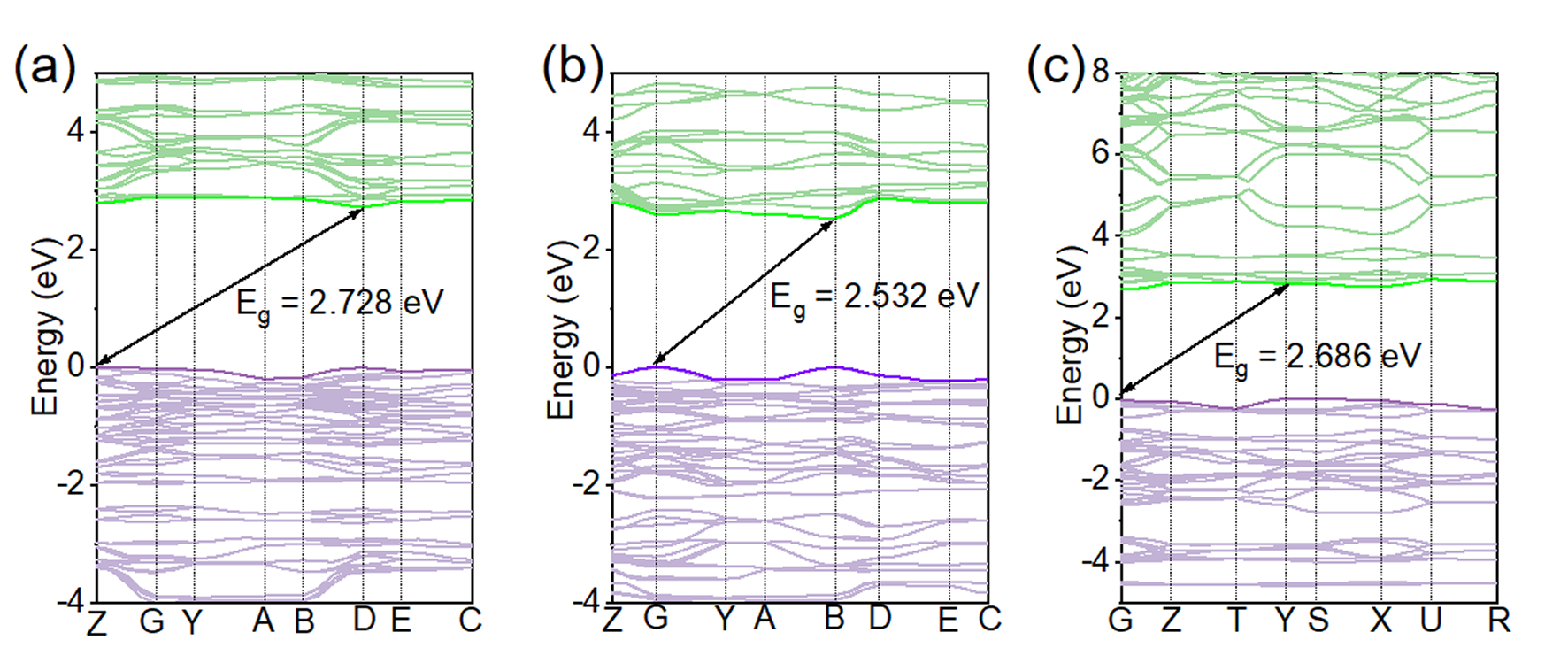


**Figure S32.** Calculated bandgaps of **XOF-1** (a), **Hybrid-XOF-1** (b) and **XB-HOF-1** (c).


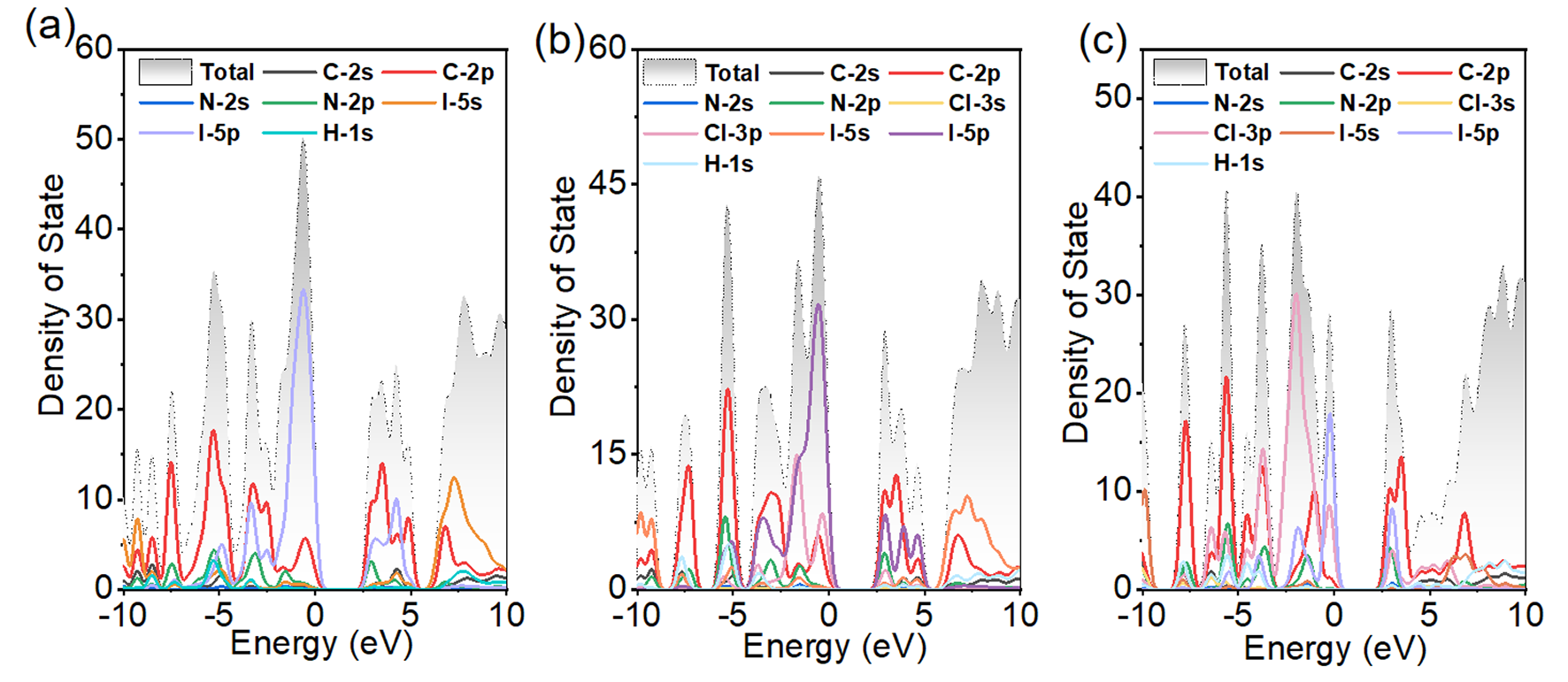


**Figure S33.** The PDOS of **XOF-1** (a), **Hybrid-XOF-1** (b) and **XB-HOF-1** (c).

**Reference**:

1. Blessing, R. H., An Empirical Correction for Absorption Anisotropy. *Acta Crystallogr A* **1995,** *51*, 31-38.

2. Sheldrick, G., Crystal structure refinement with SHELXL. *Acta Crystallogr. Sect. C* **2015,** *71* (1), 3-8.

3. Spek, A. L., Single-crystal structure validation with the program PLATON. **2003,** *36*, 7-13.

4. Munk, P. K. F., An Article on Optics of Paint Layers. *Z. Tech. Physical* **1931,** *12*.

5. Cao, L.; Peng, G.; Liao, W.; Yan, T.; Long, X.; Ye, N., A microcrystal method for the measurement of birefringence. *CrystEngComm* **2020,** *22* (11), 1956-1961.

6. Huang, W.; Zhang, X.; Li, Y.; Zhou, Y.; Chen, X.; Li, X.; Wu, F.; Hong, M.; Luo, J.; Zhao, S., A Hybrid Halide Perovskite Birefringent Crystal. *Angew. Chem. Int. Ed.* **2022,** *61* (22), e202202746.

7. Segall, M. D.; Philip, J. D. L.; Probert, M. J.; Pickard, C. J.; Hasnip, P. J.; Clark, S. J.; Payne, M. C., First-principles simulation: ideas, illustrations and the CASTEP code. *J. Phys.: Condens. Matter* **2002,** *14* (11), 2717.

8. Milman, V.; Winkler, B.; White, J. A.; Pickard, C. J.; Payne, M. C.; Akhmatskaya, E. V.; Nobes, R. H., Electronic structure, properties, and phase stability of inorganic crystals: A pseudopotential plane-wave study. *International Journal of Quantum Chemistry* **2000,** *77* (5), 895-910.

9. Perdew, J. P.; Burke, K.; Ernzerhof, M., Generalized Gradient Approximation Made Simple. *Phys. Rev. Lett.* **1996,** *77* (18), 3865-3868.

10. Lin, J. S.; Qteish, A.; Payne, M. C.; Heine, V., Optimized and transferable nonlocal separable ab initio pseudopotentials. *Phys. Rev. B* **1993,** *47* (8), 4174-4180.

11. Frisch, M. J.; Trucks, G. W.; Schlegel, H. B.; Scuseria, G. E.; Robb, M. A.; Cheeseman, J. R.; Scalmani, G.; Barone, V.; B., M.; Petersson, G., Gaussian 09, Revision C.01, Gaussian, Inc., Wallingford, CT. **2010**.

12. Lu, T.; Chen, F., Multiwfn: A multifunctional wavefunction analyzer. *J Comput Chem* **2011,** *33* (5), 580-592.

13. Lu, T.; Chen, Q., Independent gradient model based on Hirshfeld partition: A new method for visual study of interactions in chemical systems. *J Comput Chem* **2022,** *43* (8), 539-555.

14. Qi, L.; Jiang, X.; Duanmu, K.; Wu, C.; Lin, Z.; Huang, Z.; Humphrey, M. G.; Zhang, C., Record Second-Harmonic Generation and Birefringence in an Ultraviolet Antimonate by Bond Engineering. *J. Am. Chem. Soc.* **2024,** *146* (14), 9975-9983.

15. Li, Y.; Ok, K. M., Crystal clear: unveiling giant birefringence in organic–inorganic cocrystals. *Chem. Sci.* **2024,** *15* (26), 10193-10199.

16. Li, Y.; Lee, J.; Ok, K. M., Designed Metal-Free Quasi-1D Crystals with Giant Birefringence. *Chem.-Eur. J.* **2025,** *31* (28), e202500849.

17. Li, J.-J.; Xu, M.-B.; Wang, M.-C.; Lin, Z.; Lin, Y.; Hu, Y.-X.; Lian, J.-M.; Chen, J.; Du, K.-Z., Breaking the Birefringence Barrier in B–O Crystals via B–C π-scaffolding. *J. Am. Chem. Soc.* **2025,** *147* (33), 29864-29874.

18. Guo, P.-H.; Zhang, X.; Guo, Y.; Zhang, N.; Chen, Y.-G.; Jiang, X.; Lin, Z.; Zhang, X.-M., Giant Birefringence and Solar-Blind Ultraviolet Transmission by Coordination-Induced Assembly of π-Conjugated Pyridine–Carboxylate. *Angew. Chem. Int. Ed.* **2025,** *64* (12), e202508997.

19. Chen, C. A.; Li, Y.; Huang, H.; Jin, C.; Zhang, B.; Ok, K. M., Giant Birefringence Enabled by the Highly Anisotropic Linear IX_2_^−^ (X = Cl, Br) Building Blocks. *Angew. Chem. Int. Ed.* **2025,** *64* (27), e202506625.

20. Chen, Q.-Q.; Hu, C.-L.; Zhang, M.-Z.; Mao, J.-G., (C_5_H_6.16_N_2_Cl_0.84_)(IO_2_Cl_2_): a birefringent crystal featuring unprecedented (IO_2_Cl_2_)^−^ anions and π-conjugated organic cations. *Chem. Sci.* **2023,** *14* (48), 14302-14307.

21. Chen, J.; Xu, M.-B.; Wu, H.-Y.; Wu, J.-Y.; Du, K.-Z., Halogen Bond Unlocks Ultra-High Birefringence. *Angew. Chem. Int. Ed.* **2024,** *63* (44), e202411503.

22. Zhang, P.; Dong, X.; Huang, L.; Lin, Z.; Zhou, Y.; Zou, G., Double Sb-N Coordination Enables Record-High Birefringence in UV-Transparent Crystals. *Angew. Chem. Int. Ed.* **2025,** *64* (21), e202424756.

23. Zhang, P.; Dong, X.; Huang, L.; Lin, Z.; Zhou, Y.; Zou, G., Dual-Sided Multidentate Coordination Strategy Enables Record Birefringence in UV-Transparent Antimony-Based Hybrid Crystals. *Angew. Chem. Int. Ed.* **2025,** *64* (36), e202513511.
